# A Review on the Stability Challenges of Advanced Biologic Therapeutics

**DOI:** 10.3390/pharmaceutics17050550

**Published:** 2025-04-23

**Authors:** Sruthi Sarvepalli, Shashank Reddy Pasika, Vartika Verma, Anusha Thumma, Sandeep Bolla, Pavan Kumar Nukala, Arun Butreddy, Pradeep Kumar Bolla

**Affiliations:** 1 College of Pharmacy and Health Sciences, St John’s University, Queens, New York, NY 11439, USA; sruthi.sarvepalli@gmail.com (S.S.); drpavankumarnukala@gmail.com (P.K.N.); 2 Department of Biotechnology, National Institute of Pharmaceutical Education and Research—Raebareli (NIPER-R), Raebareli 226002, India; shashankreddy318@gmail.com; 3 Laboratory of Translational Research in Nanomedicines, Lifecare Innovations Private Limited, Lucknow 226021, India; vartikaverma256@gmail.com; 4 Department of Pharmaceutical Sciences, College of Pharmacy, Nova Southeastern University, Fort Lauderdale, FL 33328, USA; 5 Department of Statistical Programming, Fortrea, Durham, NC 27709, USA; sandeepbolla@gmail.com; 6 Department of Pharmaceutics and Drug Delivery, School of Pharmacy, The University of Mississippi, University, MS 38677, USA; 7 Department of Biomedical Engineering, College of Engineering, The University of Texas at El Paso, El Paso, TX 79968, USA

**Keywords:** biologics, stability concerns, stabilization strategies, mRNA lipid nanoparticles, cell therapy, monoclonal antibodies, fusion proteins, conformational instability, colloidal instability, gene therapy, antibody–drug conjugates (ADCs), stabilization mechanisms, non-viral vectors, viral vectors

## Abstract

Advanced biotherapeutic systems such as gene therapy, mRNA lipid nanoparticles, antibody–drug conjugates, fusion proteins, and cell therapy have proven to be promising platforms for delivering targeted biologic therapeutics. Preserving the intrinsic stability of these advanced therapeutics is essential to maintain their innate structure, functionality, and shelf life. Nevertheless, various challenges and obstacles arise during formulation development and throughout the storage period due to their complex nature and sensitivity to various stress factors. Key stability concerns include physical degradation and chemical instability due to various factors such as fluctuations in pH and temperature, which results in conformational and colloidal instabilities of the biologics, adversely affecting their quality and therapeutic efficacy. This review emphasizes key stability issues associated with these advanced biotherapeutic systems and approaches to identify and overcome them. In gene therapy, the brittleness of viral vectors and gene encapsulation limits their stability, requiring the use of stabilizers, excipients, and lyophilization. Keeping cells viable throughout the whole cell therapy process, from culture to final formulation, is still a major difficulty. In mRNA therapeutics, stabilization strategies such as the optimization of mRNA nucleotides and lipid compositions are used to address the instability of both the mRNA and lipid nanoparticles. Monoclonal antibodies are colloidally and conformationally unstable. Hence, buffers and stabilizers are useful to maintain stability. Although fusion proteins and monoclonal antibodies share structural similarities, they show a similar pattern of instability. Antibody–drug conjugates possess issues with conjugation and linker stability. This review outlines the stability issues associated with advanced biotherapeutics and provides insights into the approaches to address these challenges.

## 1. Introduction

Advanced biologic therapeutics have significantly transformed the healthcare landscape by providing effective treatments for various diseases, including cancer, genetic disorders, and autoimmune conditions [1]. Advanced biologic therapeutics include gene therapy, cell therapy, mRNA therapy, and others. In contrast to conventional small molecules, biologic therapies contain complex macromolecules, such as genes, proteins, cells, nucleic acids, etc., that require complex characterization techniques [2,3]. Additionally, the delivery of biologics also poses substantial stability challenges due to their inherent susceptibility towards physical and chemical degradation [4,5].

Among the various biologic therapeutics, gene therapy has emerged as a transformative approach aimed at correcting genetic defects by delivering functional genes into a patient’s cells. It commonly employs viral vectors, such as adeno-associated viruses (AAVs) or lentiviruses, to introduce genetic material into the target cells. Non-viral methods such as lipid nanoparticles (LNPs) are also being explored for gene therapy. However, the stability of gene therapy products is of critical concern. Viral vectors are prone to degradation during storage, which can impact their therapeutic efficacy [6]. Cell therapy involves the transfer of autologous or allogenic cells into the patient for therapeutic purposes [7]. The challenge with cell therapy is maintaining the viability and stability of cells from production to administration. Additionally, cell therapies require cryopreservation and the use of cryoprotectants to ensure that the cells remain active and functional. Maintaining stability is found to be crucial, as cell viability is directly linked to therapeutic success [8]. On the other hand, messenger RNA (mRNA) therapies involve the use of mRNA, which, upon administration, produces specific proteins. LNPs are frequently used as delivery systems for this type of therapy. Both LNPs and mRNA are prone to degradation, thus requiring excipients to maintain their stability [9,10].

Monoclonal antibodies (mAbs) are proteins that target specific antigens and are often administered intravenously or subcutaneously. mAbs are prone to stability issues such as conformational (due to altered protein structure) or colloidal instability, which frequently lead to aggregation [11]. Antibody–drug conjugates (ADCs) are composed of drugs that are chemically attached to mAbs and which specifically target cancer cells [12]. However, the stability of ADCs is critically dependent on the integrity of the chemical linker between the mAb and the drug (payload). This is because the conjugation changes the properties of the attached mAb [13]. Instability in the linker can result in the premature release of the cytotoxic agent, thus leading to reduced therapeutic efficacy and increased toxicity [14,15].

**Figure 1:**
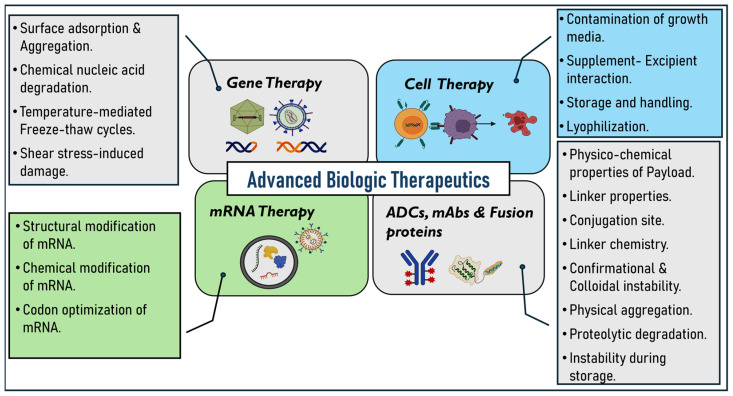
Instability challenges related to advanced biologic therapeutics.

On the other hand, fusion proteins are prepared by combining two or more genes that can encode different proteins. They contain two or more different protein domains in a single molecule [16]. These proteins will have unique stability issues due to the complexity of their structures [17,18]. Given this background, it is evident that advanced biologic therapies are susceptible to stability issues. This review aims to present a detailed examination of the stability challenges associated with these advanced biologic therapeutics, including gene therapy, cell therapy, mRNA therapies, mAbs, ADCs, and fusion proteins (Figure 1). Additionally, we emphasize the various strategies and approaches discussed and employed in the literature, such as the use of novel excipients, lyophilization techniques, optimized linkers, and conjugation methods to address these challenges and ensure the stability and efficacy of these therapeutics throughout development and clinical use.

## 2. Stability Considerations for Advanced Biologic Therapeutics

### 2.1. Gene Therapy

**Figure 2:**
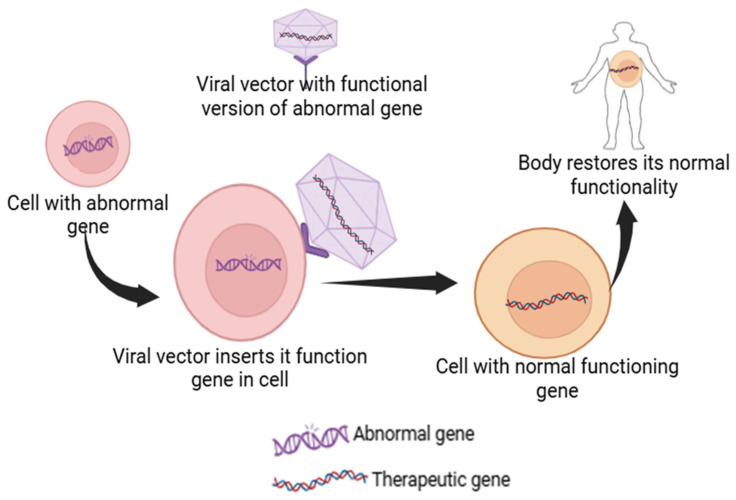
Schematic representation of a viral vector inserting its functional gene into the abnormal gene-containing cell to restore its native body function.

Gene therapy is an advanced medical technique that uses deoxy ribonucleic acid (DNA) recombination and gene cloning to treat or prevent genetic problems. This technique has the potential to directly repair or substitute genes that cause genetic conditions such as cancers, cystic fibrosis, acquired immunodeficiency syndrome, cardiovascular ailments, and sickle cell anemia. Gene therapy offers a direct approach to targeting the underlying genetic condition by addressing the root cause, offering a potentially curative solution compared to the traditional treatments that focus on alleviating symptoms. For example, Leber congenital amaurosis, a rare eye disorder, is caused by a mutated RPE65 gene and results in inherited retinal dystrophy. This condition can be treated with gene therapy using a product called Luxturna to deliver a healthy version of the gene to the retinal cells. Similarly, sickle cell disease and beta-thalassemia are caused by heritable, single-gene mutations, leading to complications such as anemia and reduced hemoglobin production, respectively. CRISPR-Cas9-based gene therapies such as CTX001 can be used as a treatment to boost the production of healthy fetal hemoglobin, thereby addressing the root cause and reducing the need for blood transfusions [19,20,21,22]. Gene therapies usually involve the delivery of a functional gene into the cells of the patient, which can either correct a mutation, replace the faulty gene, or help fight a disease (Figure 2). This is accomplished by using vectors, with viral vectors being most frequently used due to their capability to proficiently enter cells. Viral vectors in gene therapy come with limitations such as immunogenicity, insertional mutagenesis, and potential toxicity to host cells [23]. Non-viral methods like nanoparticles offer advantages such as reducing the risk of triggering an immune response and avoiding the integration of genetic material into the host genome, thus enhancing safety. For example, CRISPR-Cas9 can be delivered via non-viral methods like LNPs or electroporation, allowing for targeted gene editing without the risks associated with viral vectors [24].

Gene therapy has already experienced success in clinical trials and has been introduced into the pharmaceutical market with hope for patients with conditions such as metachromatic leukodystrophy, Leber congenital amaurosis, spinal muscular atrophy, sickle cell disease, β-thalassemia, and severe combined immunodeficiency [24]. However, some challenges are associated with gene therapy, which include its off-target effects, cost, and immune response. Moreover, a risk–benefit analysis should be performed to ensure the safety of the treatment [25,26].

#### 2.1.1. Viral Vectors

Gene therapy requires vectors to transfer genetic material effectively and safely into host cells. These are precisely designed to target and enter specific cells, penetrate their membranes, and become an integral part of the genetic material, ensuring the unremitting production of the therapeutic genes. Optimal vector design is indeed essential for achieving favorable gene therapy, with the stability aspects of DNA being a critical consideration. Naked nucleic acids are prone to degradation by nucleases, possess challenges in cellular uptake, and may trigger immune responses, limiting their therapeutic efficacy. Therefore, the optimization of thoughtful vector engineering can improve translation efficiency, refine tissue specificity through promoter and regulatory region optimization, reduce immune response activation, and increase manufacturing yield and quality [27,28]. The selection criteria of viral vectors are key attributes and depend on various factors, such as efficiency, ease of production, safety, toxicity, and stability [29]. The primary categories of viral vectors comprise retroviruses, adenoviruses, adeno-associated viruses, lentiviruses, and herpes simplex viruses, each with distinct characteristics [30].

##### Adenovirus Vectors (Ad-Based Vectors)

Adenoviral vectors are non-enveloped, double-stranded DNA viruses, widely used in gene therapy due to their high transduction efficiency and ease of scalability. However, a significant challenge with these vectors is their potential to trigger immunological reactions, stemming from pre-existing immunity and adaptive immune responses [31,32]. First-generation vectors can accommodate transgenes up to 6.5 kb but still tend to elicit immunological responses. Second-generation vectors were designed to address the shortcomings of their predecessors by minimizing the expression of viral genes, thereby reducing immunogenicity and further enhancing transgene capacity. Third-generation vectors, capable of holding up to 36 kb of genetic material, offer reduced chances of immune responses and longer sustainability, although they require a helper virus to function properly [33,34,35,36]. A total of 50% of the human gene therapy research worldwide utilizes Ad-based vectors for the development of new treatments [28,37]. However, these vectors enhance inflammatory reactions and do not integrate into the host’s chromosome. Hence, they are better suited for applications that require short-term gene expression, such as the delivery of suicide genes in cancer research [38].

##### Retrovirus Vectors

Retroviruses encapsulate two copies of positive-strand RNA within their structure. They have gag, pol, and env genes, surrounded by long terminal repeats (LTRs) that act as enhancers and promoters [39]. They are employed in gene therapy due to their ability to integrate transgenes into the genome of the host cells, resulting in sustained expression for an extended period [40]. However, the major limitation of the retrovirus vector includes induced insertional oncogenesis due to random genomic integration and a failure to transduce non-dividing cells [41]. They are used for transferring genes into rapidly dividing cells over an extended period. However, the integration process in the host cell poses certain challenges, like insertional mutagenesis, oncogene activation, immune response, and the dysregulation of host oncogenes [42,43].

##### Adeno-Associated Virus Vector (AAV)

AAV vectors are used as delivery vehicles due to their safety and effectiveness. They are harmless, non-replicating viruses that can effectively transport therapeutic genes to specific cells and tissues, resulting in sustained gene expression [29]. They have several advantages, such as a wide range of tissue targeting, a minimal immune response, and non-integration into the host genome, minimizing genetic damage. However, they have certain challenges, including pre-existing immunity, restricted cargo capacity, and manufacturing cost [44]. There are three approved AAV-based gene therapies for retinal dystrophy, spinal muscular atrophy, and hemophilia B [45].

##### *Herpes Simplex* Virus Vector (HSV)

HSV vectors are large, enveloped viruses classified within the *Alphaherpes* virinae subfamily of the Herpesviridae family. HSV-1-based vectors can infect proliferating and non-dividing cells, without integrating into the genome of the host cells [46]. They are considered a highly promising technique for delivering genes to the central nervous system (CNS). Additionally, HSV vectors have been used for the treatment of recurrent breast cancer, head and neck cancer, unresectable pancreatic cancer, refractory superficial cancer, melanoma, and various neurological and pain problems. Various studies have demonstrated HSV as a possible remedy for delivering genes to the CNS and other regions where conventional gene therapy vectors may be inadequate [47,48].

#### 2.1.2. Stability Issues Associated with Gene Therapy

Developing a gene-based medication poses significant stability challenges that can impact its safety and efficacy [49]. The different physical and chemical factors that affect the stability of gene-based therapeutics are enumerated below.

##### Aggregation

Aggregation hinders the effectiveness and safety of viral vectors. AAVs and lentiviruses can undergo aggregation during manufacturing and storage, forming larger complexes that cannot effectively enter into host cell, thereby reducing the efficacy of therapy [50,51]. Aggregation is caused due to factors such as temperature, stress, and pH, often caused by the destabilization of the capsid, leading to the poor therapeutic efficacy of the vector. Moreover, aggregation of the viral vectors can cause poor quality control and may elicit a stronger immune response, causing a severe inflammatory response in patients [42,52].

##### Adsorption

The adsorption (unwanted binding) of viral vectors on the surface can lead to a significantly decreased efficiency and therapeutic outcome of the gene delivery. Various viral vectors tend to adsorb on various surfaces such as cellular membranes, container material, and purification media during the manufacturing and storage process [53,54]. This can lead to decreased transduction efficiency. Adsorption can cause conformational changes in the viral vector that may compromise the integrity and rate of infectivity [55]. This further complicates the purification process, leading to the uneven distribution of full versus empty capsids, affecting the overall quality of a final product [56].

##### Deamidation, Oxidation, and Hydrolysis

Deamidation, oxidation, and hydrolysis are amongst the other critical factors that significantly affect the stability and efficacy of a gene therapy product. These can cause altered immunogenicity and an increased risk of adverse events in patients. Deamidation is the most common post-translational modification that can affect the structural integrity of the vector, leading to reduced effectiveness, and can cause altered immunogenicity, as the deamination of proteins may present different epitopes to the immune system, potentially causing unintended immune responses [57,58]. Oxidation can be detrimental to the viral vectors as it can enhance the production of reactive oxygen species, damaging the viral protein and affecting the stability and functionality. Hydrolysis can also impact capsid stability and cause the premature release of genetic material before it reaches target cells, thereby reducing therapeutic efficiency. Overall, the chemical process imposes manufacturing complexity and affects the quality of the product [59,60].

##### Freeze–Thaw (F/T)

The freeze–thaw process also poses a significant challenge to the stability of AAVs used in gene therapy. AAVs are sensitive to environmental conditions, and uncontrolled handling can lead to degradation, loss of infectivity, and reduced therapeutic efficacy. The mechanisms of instability during freeze–thaw include capsid rupture, DNA release, and aggregation. Freeze–thaw cycles can lead to the rupture of the capsid, which compromises its structural integrity, which will increase the risk of non-encapsulated DNA release [61,62]. A study found that adding ≥0.0005% *w*/*v* of poloxamer 188 (P188) eliminated substantial recovery losses (up to ~60% without P188) and minimized rupture to ≤1% per freeze–thaw cycle. Utilizing controlled-rate freezing and thawing cycles can help to ensure the reliability and success of applications with viral vectors [62].

##### Shear Stress

Shear stress, a force exerted by a fluid on a cell, is observed with gene therapy products that impose a major challenge on the stability [63]. The major production steps that can cause shear stress are caused by agitation, aeration, continuous gassing, and the few microfluid platforms that are used to study cellular responses [64]. It can lead to the aggregation or denaturation of viral capsids, compromising capsid stability and transduction efficiency. The aggregation caused due to shear stress can cause functional losses of vectors, causing altered pharmacokinetics and biodistribution upon administration [23].

##### Temperature

Temperature plays a critical role in the stability of gene therapy products, with both high and low temperatures posing significant challenges. Variations in the temperature can significantly affect the behavior of these vectors [65,66]. Exposing frozen gene therapy products to ambient temperatures during the transfer from manufacturing to shipping can cause severe thermal shock. This can lead to the denaturation and destabilization of the viral capsid, resulting in a loss of infectivity. Temperature also influences the replication cycle of the viruses, affecting the effectiveness of gene delivery [67]. Thermal sensitivity during the manufacturing process can reduce the yield and potency of the final product. Maintaining the appropriate temperature is critical throughout the storage, transport, and handling of gene therapy products to ensure their stability and efficacy [68,69,70].

#### 2.1.3. Strategies to Address Instabilities in Gene Therapy

Various strategies are employed to address the stability issues of viral vectors to enhance their integrity, efficacy, and shelf life. Some of the approaches that can be employed to mitigate the instability issues for gene therapy are provided below.

##### Excipients

The utilization of excipients in various formulations of viral vectors is essential for addressing stability issues. They ensure the enhanced stability, efficacy, and overall performance of these biopharmaceutical products [71]. Excipients help viral particles from degrading during storage, processing, and the manufacturing process, thereby ensuring their efficacy and safety [72]. There are several types of excipients used in the preparation of gene delivery products, such as sugars, polyols, gelatine, amino acids, glycerol, and surfactants (Table 1). For example, during the development of a candidate vaccine against HIV-1 using Recombinant Human Cytomegalovirus Vector (rHCMV-1), stability was one of the major challenges. This challenge was resolved with the addition of sucrose, sorbitol, and trehalose in the viral vector formulation for stability enhancement [23]. Similarly, during the development of a formulation for the adeno-associated virus, a major challenge of stability was encountered during manufacturing and extended storage. The addition of poloxamer 188 with a low concentration of sucrose and dextrose significantly enhanced the stability of AAV vectors [62,73].

##### Lyophilization

Lyophilization is the process of removing water content from the biological product and stabilizing it without altering its functionality, thereby providing long-term storage stability. The lyophilized product [36] remains potent for long durations, ensuring its accessibility and effectiveness. For example, a modified formulation for the vaccinia recombinant vaccine (rTTV-OVA) and the adenovirus vaccine (Ad5-ENV) containing polyethylene glycol, dextran, bovine serum albumin (BSA), and L-glutamic acid (L-Glu) improved thermal stability [74].

##### Biomaterials

Biomaterials also play a significant role in addressing the stability issues in viral-based gene delivery products. They protect the viral vectors from eliciting immune responses, hence improving the delivery efficiency. For example, alginate-based hydrogels for the sustained release of AAVs show promising results in enhancing the local delivery of AAVs for gene therapy applications [75], improving stability and transduction efficiency. Similarly, chitosan nanoparticles [76] demonstrate improved stability for delivering lentivirus vector-based formulations. Poly (lactic-co-glycolic acid) (PLGA) microspheres encapsulate the adenoviral vectors [77], protecting them from degradation and resulting in the overall stability and efficacy of gene delivery [78].

**Table 1:** Summary of key stability issues and excipients used to stabilize AAVs in gene therapy.

Instability Parameter	Excipient	Stabilization Mechanism	Outcome	References
Low mass concentration of AAV	Sucrose, citrate	Increasing the glass transition temperature of the lyophilized cake	Excipients were crucial for improving the stability of AAV particles in dry formulations, hence lowering aggregation and preserving infectivity during storage.	[36,79]
Aggregation due to low ionic strength	Citrate	Providing a minimum ionic strength to inhibit aggregation	Excipients lowered particle aggregation, therefore improving formulation uniformity and transduction efficiency both during storage and usage.	[36,79]
Degradation and potency loss due to mannitol crystallization during freezing	Sucrose instead of mannitol	Avoiding crystallization-induced damage	The improved retention of viral vector integrity and potency after freeze–thaw cycles.	[36,79]
Capsid damage and genome DNA release due to over-drying	Glycerol	Preventing over-drying by increasing residual moisture content	Use of excipients resulted in reduced capsid damage, ensuring higher transduction efficiency and genome stability.	[36,79]
Short-term stability of liquid formulations	Lyophilization	Improving long-term stability at refrigerated storage conditions	Use of excipients resulted in improved long-term stability under cold storage, reducing potency loss over time.	[36,79]
Aggregation and degradation of viral vectors	Sucrose, trehalose, glycerol	Increasing glass transition temperature, maintaining structural integrity	Use of excipients resulted in enhanced stability of viral vectors, reducing aggregation and degradation while ensuring consistent therapeutic efficacy.	[80,81]
Oxidative stress and loss of potency	Antioxidants (e.g., ascorbic acid, glutathione)	Protecting against oxidative damage	Excipients aided in preserving vector potency, ensuring reliable gene delivery performance.	[80,81]
Adsorption to container surfaces	Surfactants (e.g., polysorbate 80)	Reducing surface adsorption	The addition of excipients improved the recovery of viral vectors from containers, enhancing dosing accuracy and therapeutic efficacy.	[80,81]
Thermal instability during storage	Lyophilization, spray-drying	Improving long-term stability at refrigerated or ambient temperatures	Both preservation techniques provided improved long-term stability, minimizing potency loss over extended periods.	[80,81]
Immune responses against viral vectors	Immunosuppressants (e.g., rapamycin)	Reducing vector-mediated immune reactions	Use of excipients lowered immune responses, enhancing the safety profile of AAV therapies while maintaining efficacy.	[82]

### 2.2. Cell Therapy

Cell therapies introduce a whole new paradigm in drug development. The mid-20th century saw red blood cell infusions enhance trauma, medicinal, and surgical outcomes, demonstrating cells’ transformative potential [83]. Subsequently, several cell therapies using T cells, hematopoietic stem cells, progenitor cells, and fibroblasts have received the United States Food and Drug Administration (USFDA) approval [84]. These are also currently in pre-clinical and clinical research on cell therapy for treating various diseases, including infectious diseases, cancer, genetic disorders, neurodegenerative diseases, and autoimmune disorders [85,86,87,88,89].

Often, the dosage forms of cell therapies are formulated as injectable suspensions either in liquid or cryopreserved form, as implantable scaffolds, and as sheets. Briefly, dosage form development involves collecting the cells from the donor and programming them, such as by treating them with artificial antigen-presenting cells or beads in a suitable media [90]. Cells collected from the patients are known as autologous cells, and developing these cells as dosage forms for the same patient is a highly controlled process. The obtained cell suspension, depending on the need, is either reinfused into the patient (for instance, CAR-T cell therapy for a cancer patient) or cryopreserved using various excipients such as buffers (tris, histidine, sodium acetate), salts (sodium chloride, potassium chloride, magnesium chloride), polymers, proteins (albumin), and preservatives and is administered through a suitable administration route [91,92]. Maintaining sterile and aseptic conditions is important during dosage form development and characterization.

#### Instability Issues of Cell Therapy and Approaches to Overcome These

Stability issues are a major challenge, specifically that of maintaining cell viability during the development and shelf life of cell-based therapies. Cell density, excipient concentrations, and potential degradation products can all impact stability. Cell therapy products are cryopreserved and stored at very low temperatures. During this process, the integrity of the cells may be damaged. Encapsulating different cells (including bacteria, fungi, plant meristems, liver and pancreatic cells, and various human cells) strengthens mammalian cell membranes, minimizes ice damage, and maintains cryopreservation, thereby increasing storage stability. The addition of cryoprotecting agents also preserves cell integrity through the vitrification mechanism. Table 2 summarizes the various stability issues with cell therapies and the approaches employed to address them.

Formulation development involves programming the cells, which can lead to alterations in the genomic stability of the cultured or processed cells [98]. The cause of this genomic heterogeneity is due to variability in cell sources. Cell therapies often use patient-derived cells or donor cells, which can have different interaction potentials with the excipients. Cell-based formulations contain different excipients such as buffers, salts, polymers, proteins, and preservatives to maintain stability or to provide physiological osmolarity. Also, during the manufacturing process, the formulation is exposed to various supplements such as process aids, which are not desired to be included in the final product. Residual levels of these supplements may interact with other excipients or cells and form unintentional products during the product’s shelf life. This will lead to batch-to-batch or supplier-to-supplier variability. To address these issues, the controlled use of excipients, evaluation of novel excipients, and use of cryopreservation media components in the manufacturing of finished medicinal products help in minimizing these issues [90,92].

Instabilities may also occur during the shipment and handling of developed cell therapy products. The transfer of frozen or growth-inhibited cells from central storage to local storage, followed by recovery, formulation, and injection to the patients, must be considered as a whole process at an early stage in the development of the product to generate an effective and efficient delivery chain to enable critical use. Use of non-conventional raw materials; condensed manufacturing timelines, for example, the vein-to-vein in 21 days approach; and contamination due to unintentional products that lead to batch-to-batch variability are some of the common issues that can raise the stability concerns of these cell therapy products [99,100,101,102]. These can be addressed by using consistent cryopreserved media or fit-for-purpose media components. Overall, the stability of cells and excipients must be evaluated in the final formulation. The stability issues can be reduced by following the appropriate measures. For instance, to avoid contamination due to unintentional products, cell culture should be standardized with fit-for-purpose media to maintain the quality. To enhance consistency and reduce batch-to-batch variation, rigorous testing protocols and advanced technologies like single-cell transcriptomics are helpful [102,103]. In addition, optimizing supply chains improves overall product consistency and reduces stability issues [92]. Also, instead of ex vivo cell engineering, developing in situ cell therapy using various biomaterials, such as lipids, polymers, inorganic materials, and others, including polyethylene glycol and peptides (e.g., CD47), as excipients holds great promise in improving stability [84]. For example, for chimeric antigen receptor T (CAR-T) cell therapy, pseudo-typing the viral particles enables the transduction of delivery systems into the specific type of immune cells. This not only avoids the expensive and complex laboratory protocols but also reduces the toxicity.

### 2.3. mRNA-Based Therapies

**Figure 3:**
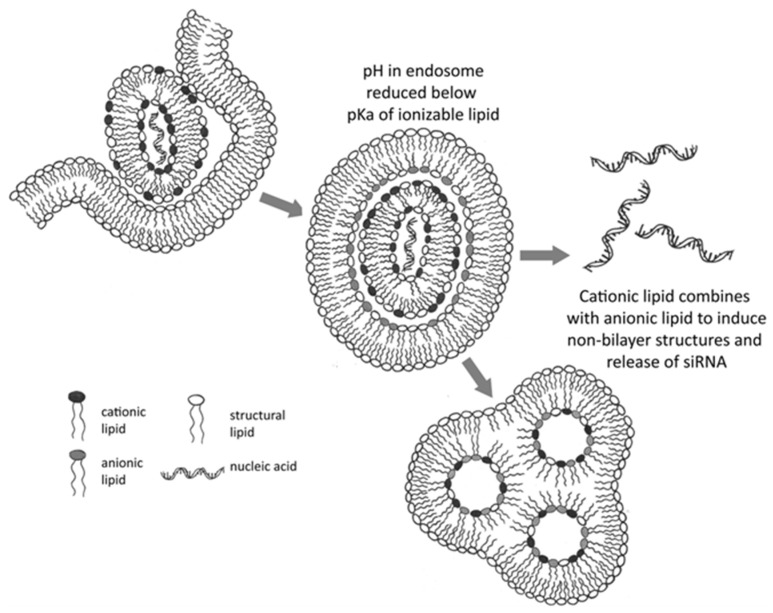
Mechanism of RNA or DNA delivery to the cytoplasm by LNPs containing ionizable cationic lipids. The acidic endosomal environment encourages lipid protonation, leading to non-bilayer phase formation and subsequent endosomal disruption, thereby facilitating mRNA escape. Adapted with permission from [110]. 2025, Anusha Thumma.

LNPs are essential delivery systems in mRNA-based therapeutics, performing critical functions such as encapsulating mRNA to shield it from enzymatic degradation and facilitating cellular uptake. A key aspect of LNP functionality is promoting endosomal escape, which is crucial for successful mRNA delivery. Following administration, LNPs are internalized into cells via endocytosis, entering endosomes [104,105]. To prevent mRNA degradation through fusion with lysosomes, mRNA must escape these vesicles. LNPs are engineered with ionizable lipids that are neutral at physiological pH but become positively charged within the acidic endosomal environment. This charge alteration destabilizes the endosomal membrane, aiding in mRNA release into the cytoplasm for translation [106,107]. This pH-dependent ionization not only protects the mRNA cargo but also enhances transfection efficiency. Additional components such as cholesterol and PEGylated lipids contribute to membrane fusion and improve colloidal stability; both of these components are vital for efficient delivery and intracellular trafficking [108,109]. The delivery process involves several stages: Initially, LNPs enclose mRNA within a lipid structure, typically using ionizable cationic lipids, which form stable complexes with mRNA’s negatively charged phosphate backbone. This encapsulation protects mRNA from extracellular RNases, enhancing its stability during circulation and storage [104,108]. Following administration, LNPs in the systemic circulation are protected by PEGylated lipids that prevent rapid clearance by the mononuclear phagocyte system, reduce aggregation, and enhance colloidal stability [110]. Subsequently, LNPs enter cells largely via clathrin-mediated endocytosis, resulting in LNP internalization within an early endosome (Figure 3). The ionizable lipids within LNPs remain relatively neutral at physiological pH to minimize toxicity during circulation. After endocytosis, the LNP-mRNA complex is contained within an endosomal compartment. As the endosome acidifies, the ionizable lipids in LNPs undergo protonation and acquire a positive charge. These positively charged lipids interact with the negatively charged endosomal membrane, disrupting its integrity through mechanisms such as the proton sponge effect and membrane destabilization, facilitating mRNA release into the cytoplasm. This interaction disrupts the endosomal membrane by promoting the formation of non-bilayer structures, leading to the release of the encapsulated mRNA into the cytoplasm for translation [109,110]. This escape is critical; failure results in mRNA degradation by lysosomes. Once in the cytoplasm, mRNA is accessible to the host cell’s ribosomal machinery and is translated into the desired protein, inducing a therapeutic or immune response. Research continues to optimize LNP composition, focusing on ionizable lipids, cholesterol derivatives, and helper lipids, to improve mRNA stability, reduce cytotoxicity, and enhance tissue-specific delivery [111,112].

#### 2.3.1. Stability Issues with mRNA-Based Therapies and Approaches to Address These Issues

**Figure 4:**
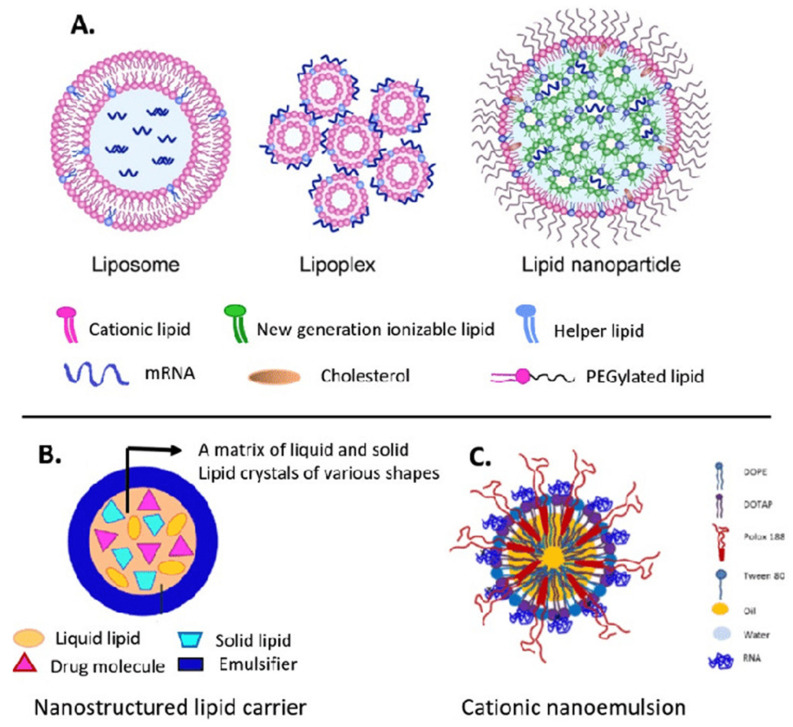
Main lipid nanocarriers of mRNA: (**A**) liposome, lipoplex, lipid nanoparticle; (**B**) nanostructured lipid carrier; and (**C**) cationic nanoemulsion. Adapted with permission from [118,119]. 2025, Shashank Reddy Pasika.

The efficient delivery of mRNA remains a significant challenge in mRNA-based therapies. Due to its large size and negative charge, mRNA molecules require specialized carriers to facilitate their entry into cells. LNPs are widely employed carriers that help in protecting mRNA from degradation, thereby favoring cellular uptake [113]. Despite their benefits, LNPs have heat resistance and target specificity issues. LNPs are inefficient in fields without cold storage because of these restrictions. This requires heat-resistant LNPs or other delivery mechanisms. Research is now targeted on the optimization of LNP composition to improve durability without compromising mRNA integrity [114,115]. Advances in nanobiotechnology offer new paths to improve the overall stability and efficiency of the mRNA delivery system. Some molecular and nanotechnology methods, such as using polymeric micelles as well as particles of hybrid nano-lipoproteins, offer further protection against mRNA degradation via the use of an enzyme, i.e., RNase, that favors the overall delivery. For instance, polymeric micelles having pH-responsive cross-linked cores not only limit mRNA degradation but also allow its specific release in target cells [116]. It has been observed that cationic nano-lipoprotein particles (exhibiting high density) play a vital role in the protection of large mRNA constructs (Figure 4), as well as favoring effective in vivo expression [117].

In general, the development of more stable as well as specific targeted delivery systems plays an important role in increasing the uses of mRNA therapeutics for all over clinical applications worldwide. The stability along with the translation efficiency of mRNA used in therapeutics are highly affected by several key structural modifications including the 5′ cap, poly(A) tail, internal nucleotide substitutions, as well as secondary structure optimization that play a vital role in the protection of mRNA from degradation, enhancing protein translation, and eventually optimizing mRNA’s therapeutic potential.

##### Structural Modifications of mRNA

mRNA structural components such as the 5′ cap, ARCA, ORF, and poly(A) tail can be modified (Table 3). Anti-reverse cap analogs (ARCAs) like dimethylated guanosine dinucleotide (2mGpppG) can prevent reverse incorporation and stabilize mRNA, protecting it from degradation and promoting the translation process. In SARS-CoV-2 mRNA vaccine development, this change is crucial [120,121,122,123,124]. The literature suggests that an ideal poly(A) tail length of 100 nucleotides maximizes translation rates, while shorter tails (~75 nucleotides) promote closed-loop structures for better translation initiation and termination efficiency [125,126,127,128,129]. In therapeutic fields, adding non-adenine residues to poly(A) tails improves mRNA translation [129,130]. 2′-*O*-methylation, mainly by fibrillarin, improves mRNA stability by maintaining its integrity over time [131,132]. These changes reduce the degradation and stabilize mRNA in different cellular contexts [128,133].

**Figure 5:**
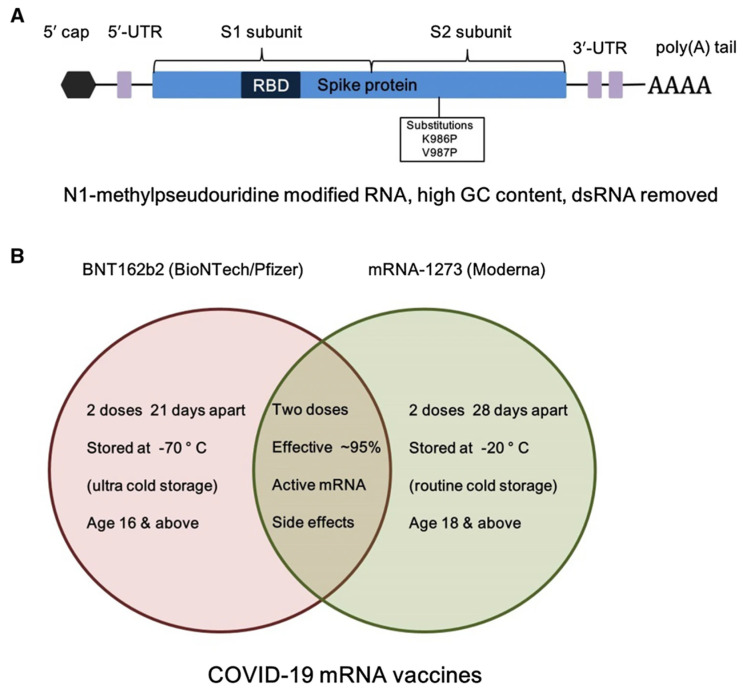
(**A**) Structural element of BNT162b2 (BioNTech/Pfizer) mRNA vaccine, and (**B**) comparison of storage and dose information of the two used BNT162b2 and mRNA-1273 COVID-19 mRNA vaccines. Adapted with permission from [124]. 2025, Shashank Reddy Pasika.

Secondary structure modifications, particularly in the form of “superfolder” mRNAs, aid in stabilizing the mRNA by making structures less susceptible to hydrolysis as well as degradation. This modification process helps in improving the half-life of mRNA for use in a variety of therapeutic modalities [134,135,136]. The encapsulation of mRNA in nanoparticles, along with combining stabilizing chemical groups as thiophosphates, suggests thermal stability as well as the efficient delivery of mRNA [128,132,137,138]. Furthermore, the optimization of codon usage, modified UTRs, and poly(A) tail modifications have also been carried out for improving mRNA stability, translation efficiency, and a achieving a faster antibody response from vaccines (Figure 5A,B) [124].

##### Chemical Modification of mRNA

Modifying these mRNA elements aids in improving the overall stability of mRNA by providing strength to its structure and enhancing its effectiveness in translation. Pseudouridine (Ψ) improves the mRNA stability via increasing base stacking interactions and maintaining RNA duplex integrity, making it less vulnerable to degradation. In this type of modification, a universal base paired with adenine, guanine, uracil, or cytosine strengthens the structure of RNA and its overall performance in different cellular environments [139,140,141]. However, this process of mRNA pseudouridylation is highly affected by nutrient deficiency, temperature stress, cellular metabolism, as well as disease states, thus affecting protein translation in various ways.

**Figure 6:**
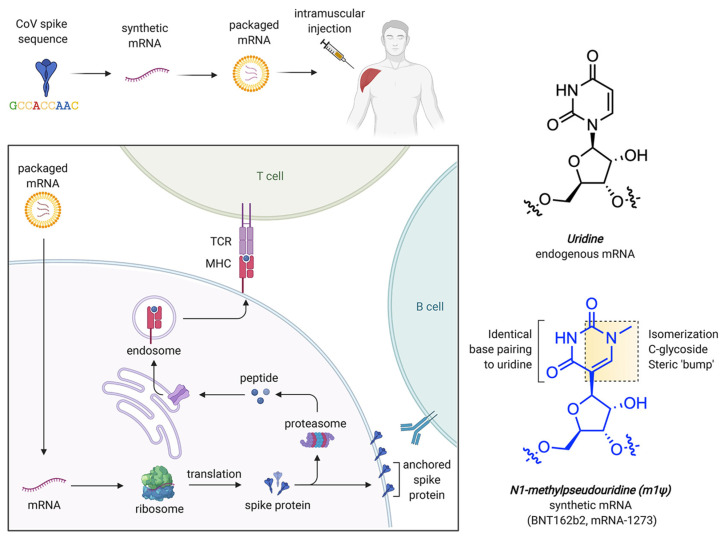
mRNA-based COVID-19 vaccine approach and structural properties of uridine and m1Ψ. Here, TCR denotes T cell receptor while MHC refers to major histocompatibility complex. Adapted with permission from [146]. 2025, Shashank Reddy Pasika.

In addition to Pseudouridine (Ψ) modification, N1-methylpseudouridine (m1Ψ) also aids in increasing mRNA stability by improving its molecular polarizability and base stacking interactions. This property makes it effective for incorporation in COVID-19 vaccines [141,142,143,144]. Computational studies have revealed that m1Ψ-modified RNA duplexes possess more stable nearest-neighbor interactions, which are crucial for secondary structure formation and for designing their therapeutic use [140,145] without causing any adverse immune reactions. The delivery carriers for this system are generally LNPs incorporated with a modified nucleobase, m1Ψ, which improves mRNA stability and decreases immune activation. Figure 6 shows the process of mRNA uptake by cells, protein synthesis, and immune response, while Figure 6 also reveals the chemical structure of modified nucleobase m1Ψ, which exchanges the natural nucleobase uracil for improving mRNA stability [146].

**Figure 7:**
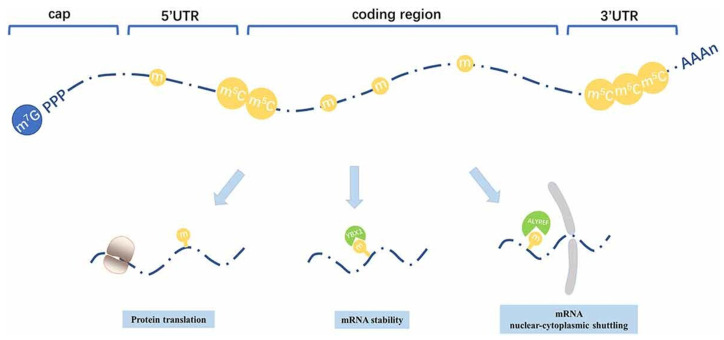
Distribution patterns and roles of 5-methylcytosine that is distributed among all regions in mRNA, primarily enriched within the 3′UTR and around the translation start sites. It helps in improving mRNA stability and its nuclear–cytoplasmic shuttling and translation. Adapted with permission from [148]. 2025, Shashank Reddy Pasika.

5-Methylcytidine (5mCyd) plays a key role in improving mRNA stability and protecting it from photodamage [146,147]. This type of RNA modification method has been widely studied across different organisms, such as mammals, plants, bacteria, as well as yeast. However, mRNA methylation is comparatively rare. NSUN2 has been identified as a key methyltransferase that is involved in mRNA methylation (Figure 7) [137,148].

##### Codon Optimization of mRNA

**Figure 8:**
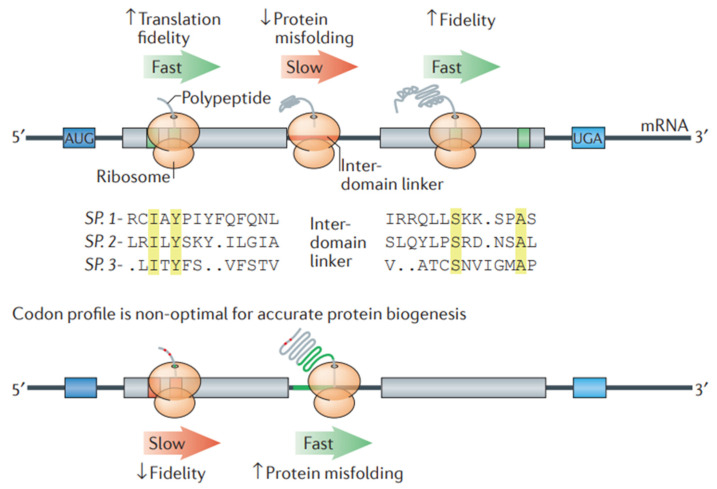
Codon optimization in a transcript helps in optimizing protein folding. A stretch of non-optimal codons (red line) can slow down the process of translation elongation. At a higher level of codon optimization (green line), protein misfolding can take place. On the contrary, ribosomes can effectively aid in translating optimal codons (green boxes) that generally encode highly conserved residues (yellow colored). Optimal codons are less susceptible to reading errors as they relate to tRNA species having a higher tRNA ratio, resulting in the high translation fidelity of the most functionally crucial residues. When amino acids are encoded via non-optimal codons (highlighted in red boxes), missense errors can take place during translation. Adapted with permission from [150]. 2025. Shashank Reddy Pasika.

For the design of stable mRNA for therapeutic purposes, codon optimization is important to improve both stability and protein synthesis by the translation of mRNA. Codon optimization increases mRNA stability, reduces the chance of translating mistakes, and reduces destruction. Additionally, it modifies the secondary structures of mRNA to avoid unstable folds that could lead to destruction. Furthermore, it guarantees effective translation rates, encouraging appropriate co-translational protein folding that improves the stability and activity of proteins. In this process of modification, the selection of synonymous codons is optimized for the host organism, thereby improving the effectiveness of mRNA-based therapies [149]. Studies have revealed that advancements in computational algorithms remarkably contributed to codon optimization efforts, suggesting the importance of computational algorithms for the development of mRNA vaccines and therapeutic proteins (Figure 8) [150]. The algorithmic method of codon optimization works by optimizing the sequence of mRNA through a balance of codon usage and structural stability. This leads to the transformation of a problem into a formal language theory model. This method showed remarkable advances in mRNA half-life and protein expression, especially in large sequences such as the SARS-CoV-2 Spike protein, which improved in vivo antibody titers in comparison to conventional benchmarks [149,151]. A model, i.e., the nearest-neighbor (NN) model, makes use of statistical physics principles for the optimization of codon usage by considering neighboring codon interactions. For effective protein expression in vivo, this model is useful, suggesting its potential for broader biotechnological usage with mRNA-based therapies [152].

It has been reported that the modification of the 3′ untranslated regions (3′ UTRs) of mRNA is a promising method for improving the stability of mRNA. The main elements of mRNA, including AU-rich regions (AREs), affect its stability via binding to RNA-binding proteins (ARE-BPs). This helps in modulating the degradation of mRNA and its translation. For example, AREs in 3′ UTRs of Ebola virus mRNAs show interactions with particular proteins, such as tristetraprolin (TTP), which help in stabilizing or destabilizing mRNA [153]. When particular dinucleotide sequences, such as TA-rich regions, are present in mRNA, RNA destabilization promotes mRNA degradation. On the other hand, when sequences are GC-rich and show interactions with protective proteins, it improves the stability of mRNA. For the design of the desired mRNA, researchers nowadays are working on manipulating these sequences [154] via the incorporation of structured viral RNA elements into mRNA constructs. This aids in improving the mRNA transcript stability as well as its expression by inhibiting the efficiency of exoribonuclease (as seen in recombinant AAV vectors) [155,156].

#### 2.3.2. Challenges and Future Directions

Codon optimization and mRNA stability engineering have improved the stability aspects of mRNA-based therapeutics; however, several limitations still remain. LNPs and polymeric nanoparticles are essential for enhancing mRNA stability, transport, and efficacy. These delivery technologies preserve mRNA against degradation, enhancing cellular absorption and tailored distribution [157,158].

LNPs protect mRNA against hydrolysis and enzymatic degradation. Nanoparticles and lipids, notably ionizable lipids, transport mRNA to target cells safely and efficiently [157,158]. These lipid particles are crucial both for effective encapsulation and for improving the endosomal release of mRNA. Cholesterol stabilizes the LNP bilayer and fuses membranes. PEG–lipids improve colloidal stability but must be tuned to prevent transfection difficulties [159]. Histidinamide-conjugated cholesterol improves endosomal escape, mRNA transport, and stability [158]. Microfluidic mixing has been employed to formulate LNPs with a consistent particle size and composition, leading to enhanced stability and delivery efficiency [160]. Regulating LNP surface charge helps in upgrading colloidal stability, particularly for inhaled delivery [161,162].

The use of the spray drying technique to powder LNPs enhances their storage stability. This SD technique has the potential to preserve mRNA functioning and eliminate cold chain storage [163]. Even tiny lipid particle contaminants like ALC-0315 affect the stability of LNPs. To maintain mRNA stability and reduce degradation, lipid particle quality must be considered [164]. Further, to improve the thermal stability of LNPs, the use of cryoprotectants, mineral encapsulation, and novel formulation approaches could be employed [165]. In the absence of cryoprotectants, LNP aggregation could occur, which may hinder protein translation [166]. Some other ways of improving the thermal stability of LNPs may include the incorporation of biomimetic minerals [167], silk fibroin [168], alcohol dilution–lyophilization techniques [169], solvent-free liquid polyplexes [170], and lyophilization. [171,172,173,174,175,176].

Polymeric nanoparticles are responsible for protecting mRNA via shielding it from degradation. Poly(amidoamine) (PAA)-based polymers in combination with chloroquinoline moieties enable endosomal escape for effective cytoplasmic release, thereby improving their therapeutic efficiency [177,178,179]. In general, the development of LNPs and polymeric nanoparticles plays a vital role in the field of mRNA delivery, stability, as well as effective immune modulation [53,180,181,182,183,184,185]. RNA-binding proteins (RBPs) and microRNAs (miRNAs) are the main regulators of mRNA stability. RBPs bind to specific RNA sequences that may protect or degrade mRNA. miRNAs, on the other hand, may target mRNA for degradation or limit translational performance. The interplay between RBPs and miRNAs is complex and essential for fine-tuning gene expression. Understanding these regulatory mechanisms is critical for developing therapeutic strategies that may target the stability of mRNA [186].

RBPs play a crucial role in cancer development and drug resistance via the regulation of stability and the translation of cancer-related transcripts [187]. RNA-binding proteins (RBPs) influence the expression of genes implicated in cancer processes like metastasis, apoptosis, and proliferation by regulating the stability of mRNA. RBP dysregulation can either destabilize tumor suppressor mRNAs or stabilize oncogenic mRNAs, which promotes the initiation and spread of cancer. In drug resistance, RBPs improve the stability of mRNAs that encode repair proteins, survival factors, or drug efflux pumps. This allows tumor cells to withstand stress and avoid treatment, which makes them viable candidates for therapeutic intervention. Engineered RBPs offer potential therapeutic uses [188]. miRNAs, via the formation of RISC complexes, prevent the translation of mRNA and also promote its degradation. The effects of miRNAs are specific; some miRNAs hinder translation, while others may promote it [189]. Codon composition can influence mRNA stability, which may be modulated via miRNAs. For example, miR-430 may reverse the stabilizing effect of optimal codons [190]. RBPs can also modulate miRNA function, affecting the stability of mRNA [190]. Modifications like methylation and acetylation may vary the structure of RNA, thereby influencing the overall mRNA decay rates [191,192]. However, more elaborate research on improved delivery systems and mRNA modification methods, and an improved understanding of interactions within biological systems will aid in the development of mRNA-based therapies.

### 2.4. Monoclonal Antibodies (mAbs)

Monoclonal antibodies have gained interest from the first approved muromonab-CD3 in 1986 to the recent approval of lecanemab in 2023, and several are currently in the clinical trial pipeline [193,194]. Intravenous administration is the most favorable approach for delivering mAbs because of instabilities in the gastrointestinal tract. The highly hydrophilic nature of protein molecules will also contribute to formulating an intravenous dosage form. Although well-versed intravenous dosages were developed, the frequent administration rates of intravenous routes paved the way for newer ones. Innovative approaches are being explored, mainly through subcutaneous routes of administration, to increase patient compliance and reduce invasiveness [195,196,197]. The subcutaneous administration of mAbs can bypass intravenous infusion with shorter administration times and is more convenient and patient centric. Currently, subcutaneous administration is a fast-growing field in mAb therapeutics, and antibodies are being formulated in high concentrations, along with matrix materials, to release the molecule for longer and maintain steady-state concentrations [197].

Commercially available intravenous monoclonal antibody formulations have buffering agents (acetate, histidine, and phosphate), tonicity modifiers (sodium chloride, sucrose), stabilizers (polysorbates), along with bulking agents/ cryoprotectants for lyophilized products [198,199,200]. The subcutaneous route is considered advantageous compared with the intravenous route, as the injection volume is limited to 2 mL for subcutaneous administration. These small volumes require highly concentrated mAbs (50–100 mg/mL), often resulting in the aggregation of proteins in the formulations. This aggregation is due to the decrease in intermolecular distances between adjacent molecules and possible electrostatic interactions. mAbs instability is particularly concerning as it may not only limit the therapeutic efficiency but may additionally possess immunogenic reactions [201]. Moreover, highly concentrated formulations exhibit increased viscosity, causing manufacturing complications. Factors including temperature, pressure, ionic strength, pH, and excipients have a positive and negative effect on the aggregation propensity in mAbs. Consequently, the formulation composition is crucial for supporting the physicochemical stability of mAbs during their manufacture, storage, and delivery [202].

The development of mAbs, stability, and structural integrity is a key aspect that needs to be investigated throughout the formulation journey. Intrinsic protein–protein cohesive interactions, the addition of external agents like excipients [203], and the processing conditions will affect the stability of the antibody in a formulation [204,205]. Mechanical stress occurs at various stages during the production and handling of mAbs (mAbs), potentially affecting their stability and efficacy [206]. Agitation stress during stirring in bioreactors and the exposure of mAbs to lower pH conditions during chromatographic purification and filtration will affect the overall stability of mAbs [207]. A study conducted by Utkarsh Tathe et al. explored instabilities related to agitation in bioreactors during production [208] and the instability that occurred by the formation of dimers and tetramers, with the effects of temperature on storage [209]. Similarly, formulations are also prone to shaking stress and aggregation during transportation, resulting in the formation of subvisible particles, leading to instabilities [210]. A comparative study conducted by Himanshu Malani et al. on the degradation of bevacizumab and trastuzumab upon different stresses, i.e., thermal stress, low pH, and shaking stress, identified aggregation mainly in thermal and chemical stress, and that could be nullified using suitable excipients [211]. The additional incorporation of surfactants in monoclonal antibody formulations mitigates the development of interfacial tension at air–liquid and solid–liquid interfaces [212], while buffering agents maintain ionic equilibrium within the solutions [213]. The aggregation is due to the conformational and colloidal instability of the proteins in the formulation. These instabilities are evaluated by the amino acid sequencing of antibodies, higher order structure analysis of proteins, purity profiles, aggregates, intact and reduced mass, potency, and binding affinity.

#### 2.4.1. Instability Issues Associated with Monoclonal Antibodies

##### Conformational Instability

The tendency of mAbs to completely unfold or partially unfold from the non-native structure in formulations leads to conformational instabilities, causing aggregation with a loss of biological activity [214]. Aggregation is an assembly of originally native and folded proteins into high molecular weight species (multimers), irrespective of their size or the nature of their peptide–peptide linkages. Hence, conformational stability is the primary contributor to protein structure; disrupting secondary and tertiary structures leads to the aggregation of proteins. Conformational instability triggers aggregation, which may arise exclusively through weak nonspecific bonds (van der Waals interactions, hydrogen bonding, and hydrophobic interactions) without altering the primary structure, a phenomenon also called self-association [215]. The formulation concerns of conformational instability include temperature stress, exposure to hydrophobic environments, and alterations in pH. The elevated protein concentrations in intravenous or subcutaneous formulation, including the surface tension between the interfaces, will affect the conformational stability.

Monoclonal antibodies are more susceptible to conformational changes due to chemical degradation by hydrolysis and deamidation. The chemical degradation is triggered by factors like exposure to light, oxidation, enzymes, and the presence of other excipients in the formulation, which are susceptible to react covalently with the primary structures of proteins [216]. Particularly, the deamination (asparagine, glutamine) and oxidation of amino acids (methionine, tryptophan) and the cleavage of disulfide bonds [217] possess instabilities. Many studies have provided significant evidence of the impact of methionine and tryptophan oxidation, along with asparagine deamidation, on the stability of mAbs [218]. Hence, there is a need for the careful screening of newer excipients apart from conventional materials and oxidation profiling [219]. Studies have reported that the addition of common peroxides in mAb formulations prevents conformational instability by inhibiting disulfide bond reduction [220,221]. Alongside the most used stabilizers like polysorbate 20, these stabilizers are also susceptible to hydrolytic degradation. The host cell proteins convert PS20 into a free fatty acid (FFA) form that can form subvisible particles over long-term storage [222]. Carle et al. studied the root cause analysis to elucidate the degradation of PS20 and PS80 and developed a mitigation strategy. This approach effectively differentiated between hydrolytic and oxidative degradation, due to increase in FFAs and non-esterified FFAs. Hence, FFA increase patterns can be used as a predictive tool for the analysis of the long-term chemical-based confirmational instability of mAbs [223].

##### Colloidal Instability

**Figure 9:**
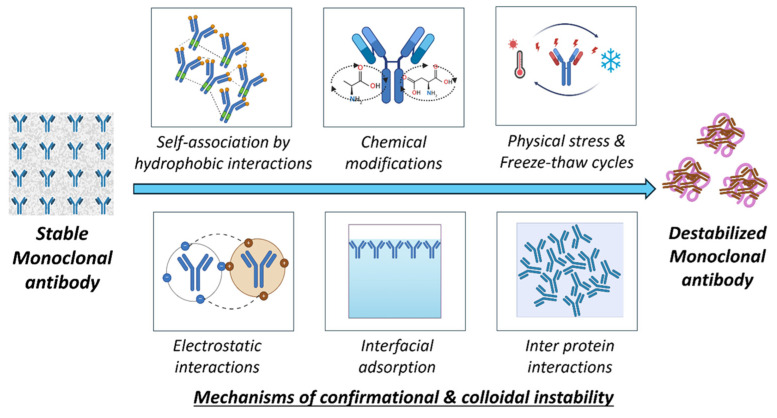
Mechanisms of conformational and colloidal instability in mAbs.

Colloidal instability occurs due to protein–protein interactions that lead to aggregation and phase separation [224]. Attractive forces like hydrophobic or van der Waals will lead to colloidal instability. The balance between these electrostatic repulsions and attractive forces in a system is crucial for maintaining the colloidal stability of mAbs in the formulation [225]. The highly concentrated mAb formulation imparts colloidal instability due to the proximity effect, i.e., individual antibody molecules are forced into closed spaces, decreasing intermolecular distances. This phenomenon adversely affects the subcutaneous delivery of mAbs, which need very high concentrations in small volumes [226]. The solution conditions, i.e., the presence of pH modifiers and ionic strength modifiers, will propagate to colloidal instability. Specifically, lower pH conditions will affect the surface charge on mAbs, resulting in a reduction in electrostatic repulsions, promoting colloidal instability. This was shown in studies performed with IgG4-N1, where colloidal instability was caused due to the unfolding of mAbs below pH 3.3 [227]. Salts affect the ionic strength in formulations; salt effects often differ based on the mAbs’ surface charge. The total impact of salt on physical stability is a balance of the salt’s interactions with water and mAbs. Salts can affect physical stability by modifying the characteristics of the mAbs–solvent system (Hofmeister effects) and by limiting electrostatic interactions (Debye–Hückel effects) [228]. A comparative study of nine mAbs demonstrated that the ionic strength affected the colloidal interactions, altering the surface charge properties. Colloidal instabilities were assessed utilizing the effective surface charge through zeta potential, diffusion interaction parameter (kD), second virial coefficient, and B_22_ [202,229]. Also, highly hydrated ions (kosmotropes) adversely influence the hydration shell of mAbs, promote hydrophobic protein–protein interactions, and detrimentally affect the protein colloidal stability (e.g., Na_2_SO_4_). This colloidal instability occurs due to the kosmotropic effect strengthening mAbs-mAbs interactions, leading to an increase in the viscosity of the formulation [230]. A comparative study was performed to assess the stability of highly concentrated mAbs; the hydroxyl group of polyethylene glycol was shown to interact with positively charged amino acid residues in mAbs. This particular binding alters the net charge of the protein and may adversely affect its colloidal stability below the isoelectric point [230,231]. This instability is due to the liquid–liquid phase separation phenomenon in which mAbs in dense fractions are separated and concentrated [231]. The chaotropic salts, including potassium iodide, diminish hydrophobic interactions by disrupting the aqueous framework surrounding solubilized molecules. They exert a colloidal impact on mAbs. However, these salts may enhance colloidal stability at low to moderate quantities [232]. The colloidal and conformational instabilities occur in a cascade fashion, and they are mostly interrelated, resulting in the aggregation and destabilization of mAbs. Figure 9 gives an overview of the mechanisms of colloidal and conformational instabilities.

##### Miscellaneous Instabilities

Photo-oxidation is also a significant concern in the growing field of mAb therapeutics. Upon light exposure, pharmaceutical proteins may change their conformation. A study by Shah et al. reported that the exposure of mAbs to light as per the ICH guidelines leads to photo-oxidation. This is due to the destabilization of two methionine groups on the CH2 domain, which is responsible for forming an aggregate [233]. Similarly, in a study conducted by Du et al., the presence of visible light impacted the stability of mAb therapeutics by the production of reactive oxygen species, leading to the oxidative degradation of tryptophan and methionine [234]. The presence of histidine-based buffers in the formulation promoted even more destabilization. Another investigation provided strong evidence that the formulation with histidine buffers increased hydrogen peroxide production due to photo-oxidation, resulting in instability [235]. The formation of oxidants in the formulation will affect conformational stability by disrupting hydrogen bonds with oxidative deamidation [236]. This promotes the exposure of hydrophobic regions, posing conformational instability. The study provides significant pieces of evidence comparing three commonly used oxidants, namely, hydrogen peroxide, tert-butyl hydroperoxide, and 2,2′-Azobis(2-amidinopropane) dihydrochloride (AAPH). The results showed that the presence of these oxidants significantly affected the stability of mAbs and promoted destabilization [220]. To mitigate oxidation-mediated instability issues, high-throughput oxidation profiling can be employed to evaluate the oxidation susceptibility of mAbs [219]. The formulation’s composition must be chosen carefully to avoid conformational and colloidal instabilities, as well as instabilities related to photochemical and mechanical stress.

#### 2.4.2. Approaches for Overcoming These Instability Challenges

##### Buffering Agents

Despite several studies on understanding mAbs’ instabilities, buffer-specific effects are consistently disregarded. Buffer composition is a critical factor in maintaining the pH and ionic equilibrium in mAb formulations. Buffer components, upon hydration, adsorb on the surface of the mAb, promoting electrostatic stabilization [213]. Tweaking these electrostatic interactions will enhance the colloidal stability of proteins. Alongside this, buffer components function as a pseudo-substrate by facilitating component binding and proton transfer [237]. Nevertheless, conventional buffers such as phosphate and succinate buffers may not always be the right choice for all mAb formulations [238]. In a study, various buffer systems were identified as alternative buffers for the stabilization effect under freeze–thaw conditions [239]. The replacement of chloride-containing buffering agents with aspartate, glutamate, acetate, sugars, and glucuronate significantly improved conformational stability [240]. The stabilization effect is due to the light-absorbing nature of buffering agents that prevent hydrolysis by obstructing activation energy. These buffering agents preserve ionic strength and enhance surface stabilization through a charge shielding mechanism, thereby enhancing colloidal stability [238]. The mAbs formulations contain chains of individual amino acids such as aspartate (pKa 3.7), glutamate (pKa 4.3), and histidine (pKa 6.5). They potentially act as buffering agents in the pharmaceutically relevant range (pH 4.0 to 7.0) [241]. The combination of histidine (50mM concentration) and arginine in formulations provides characteristic shreds of evidence for stable mAbs. In this case, histidine provides buffer capacity at a pH below the pI of the antibody, while arginine promotes increased stability and protein integrity due to chaotropic effects [242]. The chaotropic effect reduces interference with the hydrogen bonding network between water and mAb molecules and develops more intra-structural protein interactions [243]. This unique property of amino acids, preventing colloidal instability over a wide range of pH values, paved the way to explore similar excipients like arginine and glutamic acid [244]. For example, histidine with pka 6.0 and buffering capacity synergistically works with carbohydrates (sucrose, trehalose), even in freeze-drying. The native conformation of proteins is stabilized by the preferential hydration nature of carbohydrates [245]. The use of di, tri carboxylic acids (citric acid, tartaric acid) enhances the thermal stability of antibodies during freeze-drying [246]. Excipient interactions also need to be considered, as the imidazole ring on histidine accelerates the catalytic degradation of polysorbates 20 or 80 through ester hydrolysis [247]. The accumulation of fatty acids as a byproduct may induce adverse immune reactions. Therefore, multiple approaches have been provided in the literature to stabilize mAbs. Preferential molecular interactions need to be understood for the stabilization of mAbs in formulations.

##### Surfactants

The interfacial adsorption of mAbs at liquid–gas and liquid–liquid interfaces may induce structural deformation, leading to undesired colloidal and conformational instabilities. The surfactants inhibit the adsorption of monoclonal antibodies at the air–water interfaces, which often results in elevated protein concentrations at these interfaces [248]. These elevated concentrations are accountable for colloidal instabilities. This surface adsorption reveals hydrophobic areas of proteins and may also result in conformational instability [249]. Surfactants engage with exposed hydrophobic areas, inhibiting protein absorption in solutions and at the air/water interfaces [250]. Non-ionic surfactants such as PS20, PS80, and P188 are found to be effective even at lower concentrations because of their better ability to replace mAbs for adsorption at the interfaces [251]. The interfaces might be between air and water or a solid glass surface and water. The surfactant stabilization mechanism involves the formation of a protective layer on the glass surface that prevents interactions with the vial surface [252]. This stabilization effect may be due to the competitive adsorption of surfactants at the air/water interfaces, forming a monomeric layer surrounding each protein molecule. This stabilization mechanism was proved using X-ray reflectivity measurements and surface tension measurements [253]. In contrast, the gelation of mAbs at the air/water interface will also contribute to the instability. This physical gelation will be further retarded by the addition of polysorbate above the critical micellar concentration [254]. Comprehending the aggregation tendencies of various mAb–surfactant interactions and comparing them with interfacial behavior can significantly enhance the understanding of the instability process and assist in reducing aggregate formation by optimizing surfactant type and concentration in the formulation [227]. Polysorbate 80 and polysorbate 20 are the predominant surfactants utilized for stabilizing proteins in formulations. Nonetheless, challenges related to surfactants, such as contamination, degradation, and the potential induction of undesirable immunological responses, have been observed [255].

##### Viscosity Modifiers

The high-concentration mAb formulations have the potential to have high intermolecular associations. Electrostatic, hydrophobic interactions with charged molecules will contribute to these instabilities [256]. It is important to establish control over the ratio of viscosity-modifying excipients with mAbs for protein stabilization. The addition of amines such as arginine, histidine, lysine, proline, and pyridoxamine can interact with hydrophobic patches of the mAbs surface via cation–π interactions [257]. This interaction covers the hydrophobic patches on mAbs, preventing protein–protein hydrophobic associations and lowering the viscosity [258]. Another study evaluated the effect of arginine monohydrochloride, proline, and lysine monohydrochloride on the viscosity and shear-thinning ability at different high and ultra-high mAb concentrations. The outcomes stated that formulation viscosity followed the trend of arginine > proline > lysine for lowering the viscosity [259]. In a study conducted by Dear et al. [260], viscosity reduction was achieved by reducing the pH using co-solutes (camphor sulfonic acid, imidazole) and cationic amino acids (arginine, histidine, lysine), each of which contains hydrophobic groups attributed to the weakening of local anisotropic electrical and hydrophobic attractions. These electrostatic and hydrophobic attractions are responsible for network formation with increasing viscosity [260]. Charge-based (electrostatic) interactions significantly influence molecules over greater distances than hydrophobic interactions, resulting in conformational instability [261]. Sodium chloride (150 mM concentration) can reduce the viscosity of a formulation by neutralizing protein charge, hence diminishing electrostatically induced protein–protein interactions [262]. In contrast with traditional salts, newer salts like ethanolamine-DPA and diethanolamine-DPA were identified for their viscosity-reducing properties by altering hydrophobic pockets of proteins. Also, small molecules like caffeine in mAb formulations significantly reduced their viscosity. The reduction in viscosity may be due to the protein interface binding of caffeine inhibiting short-range protein–protein interactions [263]. This concludes that the careful screening of newer excipients can achieve highly stable and highly concentrated mAb formulations. Table 4 represents some of the excipients studied to enhance the stability of mAb formulations.

### 2.5. Fusion Proteins

**Figure 10:**
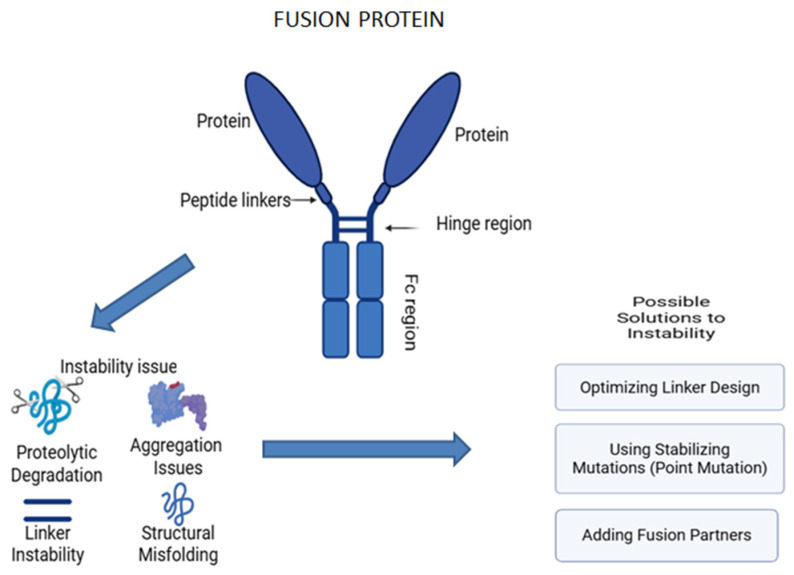
Structure and instability issues associated with fusion protein.

Fusion proteins are engineered proteins created by joining two or more genes that originally coded for separate proteins, merging the functional domains from different proteins, and enhancing their specificity and therapeutic efficiency [273]. One of the classic examples is the Fc-fusion protein, also known as a chimeric protein, which associates the Fc region of an antibody with another protein, such as enzymes or receptors [274]. This addition improves the functional characteristics of the resulting hybrid protein with advanced pharmacokinetics and therapeutic perspectives (Figure 10). Currently, they are utilized across various therapeutic fields, including cancer therapies and autoimmune disorders, due to their integrated structure and assorted mechanisms of action. The flexibility of hybrid proteins has opened new avenues and bridged the gap between diverse biological functions [275,276,277,278,279,280,281].

Recombinant technology introduces monoclonal antibodies (mAbs) alongside fusion proteins. They are both interrelated in terms of their production approaches, structural components, and therapeutic functions. The mAbs are naturally occurring, highly specific, immune molecules with long, stable half-lives, whereas fusion proteins are engineered hybrid proteins with several functional domains, having flexibility and multi-functionality [282,283].

#### 2.5.1. Instability of Fusion Proteins and Approaches to Address Stability Issues

Fusion proteins are hybrid proteins that are engineered by joining two or more proteins into one polypeptide chain. Fusion proteins and monoclonal antibodies (mAbs) have similar therapeutic applications, such as targeting specific molecules or pathways with great specificity and efficacy. However, both types of biologics encounter stability issues during production and storage, such as aggregation and structural instability. Both have comparable stabilizing mechanisms, which rely on common excipients to prevent aggregation, maintain proper folding, and enhance colloidal stability [18,281]. These instability issues arise due to heterogeneity, as fusion proteins are created through the combination of different protein domains, allowing them to target specific molecules or pathways. However, this non-natural combination can result in instability, potentially leading to aggregation. Achieving proper folding for each component in fusion proteins is a significant challenge, as combining domains that do not naturally occur together can lead to instability, such as aggregation [284,285]. The major factors that cause stability issues with fusion proteins are aggregation and protein structure breakdown, both chemically and during the storage process.

##### Aggregation

Fusion proteins contain multiple domains with variable physicochemical properties. The fusion of different domains can significantly disrupt the folding patterns. The hydrophobic regions of the fusion proteins are also exposed, causing hydrophobic interactions that lead to intermolecular interactions, facilitating aggregations of the proteins. Alterations in environmental factors like pH, stress, temperature, and concentration directly affect the stability of the protein. One of the effective ways to eliminate aggregation is to genetically fuse stabilizing peptides while preparing the fusion protein, as these maintain proper folding and mask the hydrophobic regions, thereby reducing the aggregation [286]. The addition of suitable sugars such as trehalose, mannitol, or amino acids like glycine helps in stabilizing the structural conformity of proteins by forming a protective layer [287,288].

##### Proteolytic Degradation

Fusion proteins that link the domains via flexible linkers are more sensitive to proteolytic degradation, which affects the therapeutic activity and leads to unwanted protein loss and dysfunction. Fusion proteins, during production in host cells, can be a prime target for endogenous proteases that cleave specific peptide bonds, causing the formation of non-functional proteins. The improper folding in the fusion protein makes it more susceptible to degradation by the ubiquitin–proteasome system. The utilization of genetically modified host cells that lack or have reduced specific protease activity can be a way to minimize proteolytic damage. The addition of protease inhibitors like MG132 during cell cultures will also prevent degradation by inhibiting the host-cell-derived proteases. Mutating the recognition sites of the known proteases in the fusion protein can prevent degradation, offering the structural stability of the fusion protein [281,289,290,291].

##### Instability During Storage

During the storage process, fusion proteins, especially in liquid formulations, are unstable due to multiple freeze–thaw cycles, temperature fluctuations, and inappropriate buffer conditions. This leads to unfolding, aggregation, or degradation over time, leading to a loss in protein efficacy. Therefore, lyophilization can be an effective way to tackle these instability issues [4]. The removal of water content provides a lower risk of microbial growth and degradation. Utilizing various cryoprotectants like mannitol, sucrose, or trehalose provides a protective glassy matrix to the formulation, preventing further damage [281]. Understanding the mechanism behind the instability issue can aid in choosing the proper targeting strategies to enhance the stability of the fusion protein, thereby increasing the therapeutic efficacy [291,292].

### 2.6. Antibody–Drug Conjugates (ADCs)

Antibody–drug conjugates are characterized as targeted therapeutics, sustaining a steady increase in the biopharmaceutical sector due to the increasing requirement for combination therapies. These ADCs exhibit stability concerns related to storage and in vivo conditions. The instabilities arise from high-dose formulations and combination conjugates developed by attaching highly potent small molecules to mAbs. The first ADC developed was Mylotarg (gemtuzumab ozogamcin), approved in the year 2000 for the treatment of myeloid leukemia. Moreover, 15 FDA-approved ADCs are currently available on the market up to October 2023, and several hundreds are in clinical trials [293,294]. A typical ADC will have an mAb and a payload molecule linked with a chemical linker. The payloads are 100–1000 times more potent than small molecules, can be termed “warheads“, and are the final effector component of ADCs. These payloads are inhibiting transcription, targeting DNA (duocarmycins, calicheamicins, pyrrolobenzodiazepines, irinotecan), or inhibiting tubulin (auristatin monomethyl auristatin E, monomethyl auristatin F) or maytansinoids [295]. The prerequisites for selecting these payloads include solubility, stability, high conjugation ability, and high potency. The high potency reflects that the payload shows its cytotoxicity in nanomolar concentrations. The payload is designed to show its targeting toxicity inside the cells with a low immunogenicity and a longer half-life [296,297]. Thus, the payload should be stable and intact until it reaches the site and during cell uptake.

The payload molecules interact chemically with the linkers (cleavable and non-cleavable linkers). The cleavable linkers release the payload molecules at the tumor sites, which is mediated by the pH conditions (hydrazone linkers), glutathione concentration (disulfide linkers), and other enzymatic cleavable linkers. The other class of linkers is non-cleavable, which is only susceptible to releasing the payload molecule after enzymatic degradation of the antibody in the vicinity of the cell [298]. Examples of ADCs utilizing the cleavable mechanism include brentuximab vedotin, polatuzumab vedotin, and enfortumab vedotin with a pH-sensitive hydrazone linker. Conversely, non-cleavable linkers resist cleavage and stay intact even when attached to the lysosome. This lysozyme promotes protein degradation and releases payload molecules via cathepsin B or plasmin-mediated mechanisms. T-DM1 and mafodotin belantamab are examples of FDA-approved ADCs utilizing non-cleavable linkers [299,300].

#### 2.6.1. Instability in ADCs

Colloidal instability is one of the attributes caused by the self-aggregation of ADCs with the alteration of net charge, pI, and local charge distribution. Bioconjugation can alter the native charge on the surface of the protein when cross-linked with a linker and a payload [301]. These factors reduce the solubility of the mAbs in aqueous media, increasing the viscosity of the formulation [302]. In such formulations, long-term stability is impacted by a decrease in the repulsive forces between adjacent ADCs after conjugation. Multiple studies have investigated the impact of formulation buffers, pH, and salts influencing ionic strength in stability [303,304]. These studies provide the effects of changes in solution conditions that may influence ADCs’ stability. For instance, the addition of 0.9% NaCl retarded the colloidal stability of trastuzumab with maytansinoids’ DM1 conjugate. The instability was reported due to variations in electrostatic interactions [305].

Apart from physical stability, chemical stability is crucial to maintaining the efficacy of ADCs. The first-generation ADCs like Adcetris and Mylotarg are less stable. The stability of these ADCs is affected by the properties of the conjugation chemistry of the linker-toxin, along with the conjugation site. To mitigate these issues, second-generation ADCs like Kadcyla and Polivy were developed. The focus was on site-specific conjugation chemistry to promote homogeneity [306] and maintain linker–payload hydrophobicity. In ADCs, aggregation and fragmentation occur under environmental stress and high ionic strength environments similar to traditional mAbs. Apart from prior factors, linker stability and the breakdown of thiol bonds via conjugation pose additional instability issues [307]. Bioanalytically, instability is characterized by using various spectroscopic and physical techniques, including circular dichroism, UV-Vis, fluorescence, and dynamic light scattering. Furthermore, Differential Scanning Calorimetry (DSC) has proven to be crucial in comprehending the stability of ADCs. Size Exclusion Chromatography (SEC), hydrophobic interaction chromatography (HIC) with Ultraviolet (UV), or Multi-Angle Light Scattering (MALS) detection are necessary techniques for monitoring the existence of aggregation [308]. The antibody, linker, and payload contribute to the stability of the entire ADCs.

##### Stability Aspects with Payloads of ADCs

The hydrophobicity and charge of the payload will affect the stability of ADCs during synthesis and storage, restraining the shelf life of the formulated ADCs. They are impacted by the number of payload molecules attached to the antibody, which is called the drug-to-antibody (DRA) ratio. Guo et al. studied how a higher DRA with six payload molecules increased the hydrophobicity of the ADCs compared to DRA2-DRA4 and unconjugated antibodies in cysteine-based conjugation [308]. Another study conducted by Buecheler et al. [309] determined that the increase in DRA simultaneously retarded the stability of ADCs. The same study correlated the effect of the log *p* value of the payload molecule on aggregation by in silico modeling [309,310]. Excess conjugation with more payloads leads to the removal of disulfide bonds and a further decrease in conformational stability [311,312]. To mitigate this stability, altering the chemical structure of the payload molecule can also give positive outcomes, as the study by Johann et al. demonstrated that the negatively charged payload could exacerbate instabilities in final ADCs compared to neutral payload molecules [310]. The conjugation of relatively hydrophobic drugs to the native lysine/cysteine residues of antibodies enhances their aggregation rates. This aggregation occurs by a reduction in electrostatic repulsions and by promoting hydrophobic protein–protein interactions, leading to conformational instabilities. These interactions can be modulated by altering the ionic strength, temperature, or the presence of surfactants in the formulations [305]. In some cases, the payload imposes stability; in a case study, a developed maytansinoid-based payload showed less hydrophobicity than MMAE-based payloads. However, regardless of the drug linker and DARs, the stability was decreased with the type of payload employed [313,314].

##### Stability Aspects with Linkers in ADCs

The linker is an often-disregarded component of ADCs, which has the same importance as the payload and mAbs. The linker binds the cytotoxic payload to the antibody and ensures the stability of the ADCs, facilitating the targeted release of the payload within the tumor cell. As hydrophobic drugs (payloads) pose instabilities, there are also similar concerns in regard to linkers and antibodies. The hydrophobicity of linkers leads to the faster renal clearance of ADCs in vivo. Also, by including a hydrophobic component as a linker, the polar surface area can be decreased, hence increasing its susceptibility to colloidal association [15,315]. For example, when highly hydrophobic molecules like PBD (pyrrole benzodiazepine) are conjugated to design ADCs, they can prevent the polar areas of the antibody from interacting in a solution state and cause a loss of colloidal stability. This depends on the nature of the hydrophobicity of the linker as well as the conjugate. Moreover, the process of attaching hydrophobic components might also result in alterations in solubility [308]. PEG derivatives are among the most used linkers in targeted therapy, offering a variety of features like reducing hydrophobicity and pH regulation. Such improvements in solubility are found with the conjugation of the toxin SN38, which enhanced the solubility of ADCs by up to 23-fold due to the presence of four PEG arms [316]. Glycols provide a range of PEG linkers to assist in developing ADCs, such as PEGylated ADCs. All PEG linkers possess a purity level of over 95% and serve as the fundamental components for developing a successful ADC [317]. Two recently approved ADCs, Trodelvy and Zynlonta, were designed with a PEG moiety incorporated into their linker technology to enhance their solubility and stability [318]. In the context of PEG-based linkers, a study was conducted by AstraZeneca to stabilize ADCs with the pegylation of PBD (pyrrole benzodiazepine) using MV- MV-modified molecules. This enabled the hydrophilicity to PBD (binary complex) at the C2 position and was compared against a conventional ADC (T-C239i−SG3249), which was previously reported as being highly potent and stable. The dimerized form of macrocycle CB-8 with PEG showed more stability compared to the ADC (T-C239i−SG3249). This suggests the necessity of PEG linkers in maintaining hydrophilicity by hindering the hydrophobic pocket of the ADCs [319,320]. Another study explored the use of PEG linkers for the preparation of highly hydrophilic lysine conjugates. The comparison was made between the wide range of PEG linkers, i.e., PEG12 (MAP12PS or MAP12NP) and the PEG24 linear linker (MAL24PS). The PEG-modified linker with 12 units provided the optimum hydrophilic load for conjugation, which retarded the aggregation tendency compared to marketed Kadcyla^®^ (24 PEG units) [321]. The addition of other hydrophilic linkers like glutamic acid, disulfide linkers, and the val-cit-PAB [322] linker, and the placement and arrangement of PEG units should be carefully tuned to achieve ADCs with improved stability and pharmacokinetics.

##### Stability Aspects Concerning Conjugation Sites in ADCs

The structure and stability can be correlated with the site of conjugation for maintaining the intrinsic strength of ADCs. Eliminating a solitary interchain disulfide link results in substantial reductions in conformational stability. The site-specific cysteine conjugation in the light chain using the pyrrolobenzodiazepine linker and hydrogen-exchange mass spectrometry (HX-MS) show increased backbone stability near the conjugation site [323]. Conjugation strategies at glycated sites of antibodies may potentially improve the in vivo efficacy and strength of ADCs in animal models [324]. Also, thermal stability is influenced by site-specific conjugation, as explained by a study conducted by Kaempf et al. [325], which revealed that conjugation on the site of a highly stable CH3 domain did not lead to any conformational instability. In contrast, conjugation at the C-terminus close to the CH2 domain at L328C and T-S239C seemed to have a strong impact on the thermal stability of secukinumab [325]. Another study revealed that the lysine conjugation of trastuzumab at the CH2 domain had significantly retarded thermal stability. The stability was impacted by the increased activation energy at the CH2 domain with thermal stress [326]. It can be explained that the disulfide bonds present in the CH2 domain are responsible for partial unfolding, leading to a high aggregation propensity of ADCs. Also, the increased hydrophobic contacts and charge neutralization at these sites favor the colloidal instability of ADCs. The study was conducted using different types of mAbs targeting the effects of the conjugation site and linker on aggregation. The results suggested that the colloidal stability was influenced by the hydrophobic linker, and that the conformational stability was influenced by the conjugation site. The breakdown of a single interchain disulfide link resulted in substantial conformational instabilities of the ADC itself. The production of conjugates with a higher drug-to-antibody ratio (DAR) will further lead to the elimination of more interchain disulfide bonds and a further decrease in conformational stability, as discussed earlier [311]. To mitigate these issues, the hydrophobicity of the linker and payload conjugation can be optimized inside the buried regions of the 3D protein structure. This could be called a “Trojan horse” in the Fab region of mAbs [327]. Apart from physical instability, in vivo stability is also required to prevent the breakdown of payload and antibodies through site-specific conjugation. Transglutaminase-catalyzed isopeptide linkage showed enhanced stability in in vivo mouse models; this report specifically provided the stability of auristatin-based payloads attached to the specific site [328].

#### 2.6.2. Approaches to Overcome Instabilities

Altering the molecular nature of the antibody can increase the stability of ADCs by reducing hotspots for the chemical degradation of proteins. As mentioned earlier, site-specific conjugation has remained a pioneering approach to overcoming instability. Enhancing the glycation can potentially increase the thermal stabilization of antibodies when the payloads are attached to the C’E loop at the Fc CH2 domain [326]. Due to glycation, the masking effect observed by carbohydrate aromatic interactions with the payload at the CH2 domain enhances thermal stability [329]. Further, the data reveal a correlation between the site of conjugation, the Fc glycosylation site, and the position of the glycan with the stability of the ADC [330]. The site-directed mutagenesis and molecular dynamics simulations demonstrate that the change in Lys 207 aids the stability of the ADCs. For example, Lys 207 in trastuzumab (thiomab Tras-LCV205C). This is crucial for stabilizing linkers with acid-sensitive groups like acetals, which promote the oxidation of succinimide groups in linkers. This modification is time-consuming but very effective, and modifying the specific composition of proteins near the conjugation site can enhance the stability of ADCs [331].

The instability of linker conjugates is a major concern, as it retards the in vivo efficacy of ADCs with off-target toxicity. This off-target toxicity is due to the linker breakdown in physiological conditions, i.e., pH, isotonicity, etc. The stability is attained by modifying the linker chemistry and developing novel linkers, such as in a study which reported DNA linker-based ADCs developed with trastuzumab. The antibody was conjugated with a 37-mer oligonucleotide (ON) modified at the 5-end with MMAE (cON-MMAE) to obtain trastuzumab–DNA–MMAE [332]. The developed conjugate showed complete solubility in aqueous media and decreased the overall hydrophobicity of the toxic MMAE payload. The findings suggested an increase in cytotoxicity of trastuzumab–DNA–MMAE on HER2-negative cells, even in picogram concentrations. The conjugate remained intact for 5 days when incubated in simulated human plasma at 37 °C [332].

A study by Song et al. performed an *N*-terminal selective conjugation and showed that the conjugate was more stable than the thiol and lysine conjugation of the trastuzumab ADC. The *N*-terminal antibody conjugate was more stable in vitro and in vivo with comparatively high efficacy and low systemic toxicity [333]. Similarly, a thiol-linked antibody–drug conjugate using maleamic methyl ester was developed using cysteine-based conjugation. This method improved the in vivo stability of the ADC compared with the traditional maleimide containing unstable closed-ring thiosuccimide. Despite marketed formulations such as Adcetris, Polivy, and Padcev being developed with the traditional maleimide linking method, the former approach could be a necessary alternative for next-generation ADCs [334]. The marked in vivo stability of the linker was due to the open ring, the other group of MMAE. Furthermore, albumin solution stability was determined for the maleamic methyl ester linker (ADC mil40-12c) and the traditional marketed linker (ADC mil40-12c’) in a physiological environment. About 3.8% of the ADC mil40-12c payload was shredded compared to the traditional ADC mil40-12c, which was as high as 13.3% in 14 days. These studies provide necessary insights for developing novel linkers and linker-based chemistry to mitigate stability issues. It is important to note that among the 13 approved ADCs, 11 use cleavable linkers and 7 use peptide-based valine-citrulline-p-amino benzyloxycarbonyl (VCit-PABC) for payload delivery. Similarly, glutamic acid–valine–citrulline linkers have been explored to prevent premature cleavage in circulation as compared with valine–citrulline; the modulation in the linker gave this positive outcome [310]. This shows the importance of linkers in the stability of ADCs in vivo, and a further few linker-based stabilizations are summarized in Table 5.

**Figure 11:**
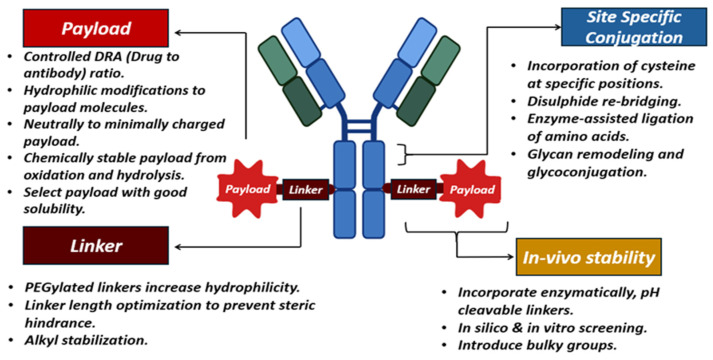
Strategies to improve the stability of ADCs.

The wide range of protein structural diversities creates the need for a distinct formulation approach. When it comes to ADCs, finding the best formulation depends mainly on linker chemistry, payload properties, and conjugation at specific sites (Figure 11). As the delivery route of ADCs is mostly via IV infusions, buffer agents and the process of lyophilization are the key aspects. The excipients, such as NaCl, dextrose, Ringer’s lactate, sodium phosphate, succinic acid, citrate, TRIS, sodium citrate, and acetate, etc., are used as diluents and buffering agents for commercial products (Bexxar™, Zevalin™, Ontak™) [341]. Developing high-concentration ADCs will have a lower risk of aggregation, because as the concentration increases, the proximity effect retards solubility with colloidal association. As the marketed IV bags are diluted before administration, this causes a decrease in stabilizer levels. The development of necessary administration techniques will eliminate these problems [342]. The excipient-mediated approach still needs to be significantly developed for ADCs, as only limited research is available. Modified excipients can exhibit enhanced physical stability and prevent conformational and colloidal instability [343]. Linker-specific stabilization can be employed for maintaining the linker drug and mAbs association in vivo (Table 5). The stability considerations with mAbs may also apply to ADCs because of their structural similarities, which were emphasized in the previous sections. Hence, there is a need for developing formulation-related approaches specific to ADCs to expedite the stability of ADCs.

## 3. Conclusions

Advanced biotherapeutics stand out as revolutionary developments, constantly changing the healthcare environment. Many diseases that were previously believed to have few treatment options may now be potentially treated with these therapies. Stability considerations for advanced biotherapeutics are complex and require phase-appropriate and matrix stability strategies owing to the limited batch sizes. A broad range of analytical tests and characterizations are crucial to determine and demonstrate the product quality profile of these biotherapeutics throughout their shelf life. Storage and handling, particularly for cells and vectors, requires additional efforts to maintain viability and activity. The presence of residual impurities such as host cell proteins and DNA affect the stability of the complex proteins (vectors and mAbs), requiring the optimization of purification strategies to enable long-term storage. Cell and gene therapy products are more sensitive to stress conditions than conventional biologics such as mAbs, making scaling up and manufacturing difficult. The growing number of approvals and the increased use of existing therapies are promising for the development of novel, advanced biotherapeutic products. Overall, considering the developments in the last decade and ongoing efforts, these advanced biotherapeutics can treat various diseases in a safe and effective way to address the patient’s unmet needs. This review highlights the major stability challenges and potential solutions for these therapeutics. While significant progress has been made, future research should focus on developing more robust delivery systems and stabilizing agents to further enhance shelf life and therapeutic performance. For instance, employing nanocarriers and advanced excipients could improve the stability of mRNA and protein-based therapies.

## Figures and Tables

**Table 2:** Summary of key stability issues with cell therapies and approaches to overcome the instabilities associated with cell therapy [93,94,95,96,97].

Instability Parameter	Approaches	Stabilization Mechanism	Outcome
Damage to cell integrity during cryopreservation leading to the formation of aggregates	Addition of cryoprotecting agents in optimum concentrations during the formulation development; Encapsulation of cells in lipids and polymers such as alginates, followed by cryopreservation.	Vitrification	A study demonstrated successful cryopreservation and recovery of large-volume alginate-encapsulated liver cell spheroids (AELSs). Encapsulation and optimized cooling and warming enabled high biomass recovery at a clinical scale, with AELSs regaining pre-freeze viability. Successful cryopreservation depends on the use of optimum concentrations of cryoprotecting agents, cooling/thawing rates, and cell-specific protocols.
Alteration of the genomic stability of cells during their programming Formation of unintentional products	In situ cell therapy with various viral (lentiviral systems) and non-viral vectors (lipids, polymers, peptides (eg: CD5, CD47)).	Pseudo-typing of viral particles for the precise transduction of the vectors or delivery systems into a specific type of immune effector cells, e.g., car-T cell therapy.	A study using non-viral vectors such as CD5-targeted LNPs successfully delivered therapeutic mRNA to lymphocytes in vivo, generating transient anti-fibrotic chimeric antigen receptor T cells that markedly enhanced cardiac function in a heart failure mouse model.
Batch-to-batch variation	Use of cryopreserved media components or fit-for-purpose media and automation throughout the development.	Maintaining the consistency and quality to reduce the variation	Development of cryopreserved media and several automation tools that enabled consistency.

**Table 3:** mRNA vaccine structural modifications that help in improving the stability and translation efficiency [124].

mRNA Structural Element	Modification	Stabilization Mechanisms	Outcomes
Untranslated regions (UTRs)	Length and structure	UTRs located at the 5′ and 3′ ends of mRNA regulate stability and translation via regulatory sequence elements and RNA-binding protein interactions. The 5′ UTR optimizes translation initiation by eliminating uORFs and non-canonical start codons, minimizing stable secondary structures that hinder ribosome recruitment, and utilizing shorter lengths to enhance translation. The 3′ UTR enhances stability and translation, with repeated sequences like β-globin 3′ UTR further boosting stability. Both the 5′ and 3′ UTRs, from cellular and viral sources, contain regions modulating mRNA fate; 3′ UTR destabilization can be application-specific.	Moderate translation efficiency
The 5′ end capping	Cap structure	The 5′ end capping, specifically with the addition of 7-methylguanosine (m7GpppN), is crucial for mRNA stability. This cap structure protects mRNA from exonuclease degradation, significantly extending its half-life. It also facilitates efficient translation by acting as a binding site for the eIF4F translation initiation complex. Utilizing anti-reverse cap analogs (ARCAs) further enhances stability and translation efficiency compared to standard caps, leading to increased protein expression and prolonged mRNA presence in cells. Essentially, 5′ capping shields mRNA from breakdown and ensures robust protein synthesis.	Improves protein synthesis and stability
Open reading frame (ORF)	Codon optimization, change in sequence	ORF stabilization of mRNA is achieved through optimized codon composition, significantly impacting translation efficiency and mRNA stability. Utilizing high GC content and matching frequent tRNA species enhances translation rates, while replacing rare codons with frequent ones speeds up translation by improving tRNA recycling. Codon optimization also includes incorporating the Kozak sequence at the start codon and modifying the stop codon, further stabilizing mRNA. Additionally, modified nucleosides like 1mΨ and m5C reduce immunogenicity and increase base pair stability, enhancing mRNA stability against degradation.	Improve protein expression
Poly(A) tail	Tail elongation	The poly(A) tail stabilizes mRNA and enhances protein translation, with its length directly correlating to mRNA longevity and translation efficiency. This stabilization is achieved by inhibiting deadenylation through the incorporation of modified nucleotides within the poly(A) tail, preventing degradation by poly(A)-specific nucleases. Optimizing the poly(A) tail length, particularly around 100 nucleotides, is crucial for controlling mRNA decay via 3′ exonucleolytic degradation. Using in vitro transcription from DNA templates allows for the precise control of poly(A) tail length, ensuring consistent mRNA stability and translation, especially vital for clinical applications.	High stability and translation efficiency

**Table 4:** Common and emerging excipients investigated for the stabilization of mAbs.

Instability Parameter	Excipients	Stabilization Mechanism	Outcome	References
Reversible self-association	Guanidine hydrochloride, trimethyl phenyl ammonium iodide, tryptophan amide hydrochloride	Lowering viscosity and weakening protein–protein interactions.	The study showed the excipient effect on reversible self-association, showing a viscosity reduction in the order of guanidine hydrochloride > trimethylphenylammonium iodide > tryptophan amide hydrochloride > ethanol.	[264]
Instability during storage	Polysorbate 20, 80, poloxamer 188	Protection of mAbs during storage against interfacial stress in the liquid state that controls visible particle formation.	Prevented the interaction of mAbs with polydimethylsiloxane, a standard container material. The resulting protein–PDSM particles were inhibited using poloxamer 188 and PSs, and superior results were obtained with polysorbates.	[252,265]
Instability during manufacturing (filtration)	Polysorbates, Brij 35	Protection of mAbs against cycloolefin copolymer and cellulose filtering components.	Polysorbates and Brij enhanced mAb stability in the presence of cyclic olefin copolymer as a model hydrophobic barrier, while poloxamer 188 showed a negligible stabilizing effect.	[266]
Instabilities with high viscosity	Aromatic amino acids, neutral dipeptide molecules-proline, polyglutamate derivatives	Excipients form electrostatic and hydrophobic interactions with mAbs, reducing aggregation.	The structural rigidity of the compounds and their aromaticity contributed to their viscosity-reducing action, which was dependent on molecular size. This study’s findings emphasize the efficacy of new proline analogs in reducing viscosity.	[258,267]
Instability with thermal stress	Third generation ionic liquids, i.e., amino acids	Reducing protein–protein interactions, altering the dielectric constant, and causing structural stabilization.	The study highlighted the use of DESs and ILs to stabilize mAbs. The non-toxic and renewable category of AA-based ILs, especially Ch-Val, excelled in preserving the structural and functional integrity of Amab, particularly at 55 °C.	[268]
Physical and chemical denaturing	HPβCD (hydroxypropyl β-cyclodextrin) in combination with polysorbates	Preventing aggregation by inhibiting protein–protein interactions and interfacial stress.	The study examined the synergistic effect of integrating polysorbates and HPβCD as excipients. Measurements of surface tension demonstrated that HPβCD improved the surface activity of polysorbates. The research indicated that the combination of these excipients can enhance the stability of mAbs in formulations.	[269]
Precipitation of highly concentrated mAbs under physiological conditions	Random hetero polymers, methyl methacrylate, isobutyl methacrylate, dimethylamino ethyl methacrylate	Altering the kinetics of precipitation with intermolecular interactions of small-molecule preservatives and mAbs.	Turbidity screening, along with hetero polymers at physiological conditions, showed improved solubilization and colloidal stability in high-concentration mAbs for SC delivery.	[270]
Subvisible particle formation of freeze-dried mAb formulations due to shaking stress	Polyol compounds like mannitol, sucrose, hydroxyethyl starch, and PS80 surfactant	Displacing water molecules around the protein shell during freeze-drying and involving hydrogen bonding.	The study found that aggregation due to shaking stress can be mitigated by adding excipients. A moisture content below 3% had little effect on the stability of freeze-dried mAbs.	[210]
Oxidative stress-induced denaturation from light and combustion of hydrogen peroxide	Taurine	Increasing physical stability. Taurine is employed to form a hydration shell around proteins, restructuring water molecules around proteins.	The study identified that even a 10 mM concentration of taurine can effectively prevent aggregation induced by light stress (365 nm) in combination with Fe^2+^ and H_2_O_2_.	[271]
Instability due to high concentration	Poly (acryloyl morpholine-co-N-isopropyl acrylamide) (MoNi)	Preferentially adsorbs to interfaces (protein–protein and protein–surfaces), thereby preventing protein aggregation.	Interfacial rheology and surface tension experiments revealed that the copolymer excipient adsorbs competitively at formulation interfaces. Additionally, the monomeric composition and preserved bioactivity of mAbs was assessed. The excipient behaved as an inactive ingredient, having no impact on the pharmacokinetic profile in mice.	[272]

**Table 5:** Various types of linkers used in the literature with their stability mechanisms in vivo.

Sr. No.	Linkers	ADC	Stability Mechanism	Outcome	References
1.	Valine–citrulline (Val-Cit) linker	Trastuzumab-Val-Cit-PAB	Stability from the enzymatic degradation of carboxylesterases and human neutrophil elastase.	ADCs developed with the exo-linker reduced premature payload release while improving the DAR, even with hydrophobic payloads, without aggregation.	[335]
2.	Val-ser (β-glc) glycoprotein linker	Trastuzumab-mavg-MMAU	Higher resistance to cleavage by lysosomal and serum enzymes, as the glycoprotein enhances the resistance to cleavage by steric stabilization.	Trastuzumab with glycopeptide linker demonstrated maleimide stabilization and enhanced resistance to cleavage in comparison to the valine–citrulline linker. The improved trastuzumab–MMAU ADC exhibits excellent pharmacokinetics in nonhuman primates.	[336]
3.	Glucuronide-modified dipeptide linker	Anti-CD79b-dipeptide-MMAE and tandem dipeptide cleavable linker	The dipeptide linkers are protected from degradation by the sterically hindering glucuronide moiety.	The results demonstrated significantly enhanced tolerance in the hematopoietic compartment, highlighting the importance of linker stability in both efficacy and tolerability. The tandem dipeptide linker outperformed the dipeptide MMAE linker.	[337]
4.	Transglutaminase-catalyzed isopeptide linkage at the C-terminal.	mAb-VC-PABC linker	Preventing degradation mediated by cathepsin B with the use of site-specific cysteine conjugation at the C-terminal of LC.	The results supported the identified position L328 as an advantageous location for cysteine conjugation, compared to the cysteine position at S239.	[325]
5.	Mono amino acid linker	mAb-monoamino linker-auristatin	The engraftment of SMDC at the Fc region of mAb significantly improves the stability of the ADC in blood circulation with cleavability in endosomal conditions.	Asn linker exhibited markedly enhanced efficacy in the cleavage of endosomal cathepsin B. This SMDC, upon conjugation with the Fc region, exhibited a significant in vivo therapeutic impact. The circulation half-life improved to 73 h and it achieved stability and anti-tumor properties.	[338]
6.	Azobenzene linker	mAb-azobenzene-MMAE	Stable in physiological conditions, and linker hydrolysis occurs in hypoxic conditions at tumor sites.	The azobenzene-based linker remained non-cleavable in healthy tissues (O_2_ > 10%), but upon exposure to the hypoxic tumor microenvironment (O_2_ < 1%), it was cleaved to release MMAE, hence, fully restoring the high cytotoxicity of the ADC.	[339]
7.	a-ammonium carbamates linkers	mAb-carbamate linker-HcyNMe dye	The zwitterionic linker reduces aggregation when compared with the Val-Ala linker	This approach improved yield and labeling density while preventing the aggregation of conjugates compared to traditional PAB linkers. The payload release was mediated by proteolytic cleavage and hypoxia-responsive nitroaryl trigger groups.	[340]

## Data Availability

Not applicable.

## Abbreviations

The following abbreviations are used in this manuscript:

3′ UTRs	3′ untranslated regions of mRNA
AAV	Adeno-associated virus vectors
ADCs	Antibody drug conjugates
CAR-T	Chimeric antigen receptor T cell therapy
CH2	Second domain of heavy chain constant regions of mAbs
DNA	Deoxyribonucleic acid
DAR	Drug to antibody ratio
Fc	Fragment crystallizable
FFAs	Free fatty acids
HPβCD	2-hydroxypropyl-β-cyclodextrin
HSV	Herpes simplex virus vectors
IgG	Immunoglobulin G
LNPs	Lipid nanoparticles
LTRs	Long terminal repeats
mAbs	Monoclonal antibodies
MMAE	Monomethyl auristatin E
PEG	Polyethylene glycol
RBPs	RNA-binding proteins
RNA	Ribonucleic acid
mRNA	Messenger ribonucleic acid
